# Tailoring Liver Transplant Decisions: How Donor–Recipient Age Matching Influences Outcomes

**DOI:** 10.1111/ctr.70477

**Published:** 2026-02-15

**Authors:** Abiha Abdullah, Berkay Demirors, Francis Spitz, Jason Mial‐Anthony, Vrishketan Sethi, Charbel Elias, Xingyu Zhang, Stalin Dharmayan, Hao Liu, Christopher Kaltenmeier, Han Shwe, Timothy Fokken, Michele Molinari

**Affiliations:** ^1^ Department of Surgery University of Pittsburgh Medical Center Pittsburgh Pennsylvania USA; ^2^ Department of Medicine, Division of Gastroenterology‐Hepatology University of Pittsburgh Pennsylvania USA; ^3^ School of Health and Rehabilitation Sciences University of Pittsburgh Pittsburgh Pennsylvania USA

**Keywords:** death‐censored graft survival, donor age, graft survival, liver transplantation, recipient age, survival

## Abstract

**Introduction:**

Donor age is a key determinant of liver transplant (LT) outcomes, but its impact varies across recipient age groups. Specific donor age thresholds associated with excess risk remain undefined.

**Methods:**

Using data from the Scientific Registry of Transplant Recipients (2011–2021; follow‐up through 2024), we analyzed first‐time, single‐organ LT recipients. Donors and recipients were stratified by age. Outcomes included patient, graft, and death‐censored graft survival. Multivariable Cox regression models adjusted for liver disease severity, comorbidities, graft type, and transplant year were used to identify donor age thresholds associated with increased risk in each recipient age group.

**Results:**

Among 70 078 recipients (median age, 57 years), mean donor age rose from 39.6 to 40.9 years (*p* =  .004), while recipient age increased from 50.7 to 51.9 years (*p* =  .003). Donor age ≥50 years was associated with a sixfold increase in mortality in pediatric recipients (aHR, 6.48; 95% CI, 1.92–21.83; *p* =  .003). For adults aged 18.1–30 years, excess mortality and graft loss were observed with donors >55 years (aHRs >2.5). In recipients aged 40.1–60 years, risk increased progressively with donor age. Among recipients ≥65 years, donor age was not significantly associated with outcomes. These thresholds were consistent across outcomes and robust in sensitivity analyses.

**Conclusions:**

This is the first national study to define recipient age‐specific donor age thresholds associated with post‐LT risk. These findings support the development of age‐informed allocation strategies and call for a reassessment of organ discard practices as donor and recipient ages continue to rise.

**Abbreviations**
ADL: Assistance for Daily Living
BMI: Body Mass Index
CIT: Cold Ischemia Time
DBD: Donation after Brain Death
DCD: Donation after Cardiocirculatory Death
HBV: Hepatitis B Virus
HCC: Hepatocellular Carcinoma
HCV: Hepatitis C Virus
HRSA: Health Resources and Services Administration
INR: International Normalized Ratio
KPS: Karnofsky Performance Scale
LDLT: Live Donor Liver Transplant
LT: Liver Transplantation
MAFLD: Metabolic Associated Fatty Liver Disease
Na‐MELD: Sodium Model for End Stage Liver Disease
PBC: Primary Biliary Cirrhosis
PSC: Primary Sclerosing Cholangitis
SRTR: Scientific Registry of Transplant Recipients
STROBE: Strengthening of the Reporting of Observational Studies in Epidemiology
U.S.: United States
WIT: Warm Ischemia Time

## 1 Introduction

In most high‐income countries, the population is aging rapidly [1, 2, 3]. In the United States (U.S.), the median age has risen from 35 in 2000 to 39 years today [2]. These demographic shifts place increasing pressure on healthcare systems [4], especially in transplantation [5].

In liver transplantation (LT), age introduces challenges on both sides of the donor–recipient equation. Older recipients often have more comorbidities, raising the risk of perioperative complications [6, 7]. At the same time, grafts from older donors are more vulnerable to ischemia‐reperfusion injury, reduced regenerative capacity, and higher rates of graft dysfunction [8, 9].

Despite these risks, donor age in not explicitly incorporated into U.S. liver allocation policies, apart from pediatric recipients. Under the current Acuity Circles (AC) model implemented by the Organ Procurement and Transplant Network (OPTN), allocation prioritizes medical urgency and geographic proximity. Age compatibility is left to the discretion of transplant teams. In practice, older organs are often declined, especially for younger recipients, over concerns about graft quality and long‐term outcomes, despite ongoing shortages [10, 11, 12, 13, 14, 15, 16, 17, 18].

Emerging evidence, however, shows that livers from older donors can yield acceptable outcomes when matched appropriately [9, 19, 20, 21, 22, 23, 24, 25]. Still, criteria for what defines a “suitable” match remain unclear. Some centers employ an “old‐for‐old” approach; others prioritize lower‐acuity candidates regardless of age. Neither strategy has been rigorously tested in contemporary national cohorts, and the age thresholds that define acceptable risk remain unknown.

As efforts to expand the donor pool and reduce waitlist mortality continue [26], a better understanding of donor‐recipient age mismatch is necessary to optimize transplant outcomes [13, 15, 24]. To support clinical decision‐making, we analyzed contemporary U.S. registry data to identify donor–recipient age differences associated with increased posttransplant risk. Our aim was to move beyond broad risk associations and define specific, actionable age thresholds to guide evidence‐based organ acceptance practices.

## 2 Methods

### 2.1 Study Design and Settings

This retrospective study used data collected in the Scientific Registry of Transplant Recipients (SRTR), a comprehensive, nationwide registry that includes detailed information on all organ donors, waitlisted candidates, and transplant recipients in the U.S. Data are submitted by members of the OPTN and maintained under the oversight of the Health Resources and Services Administration (HRSA), with additional supervision by the U.S. Department of Health and Human Services to ensure accuracy and completeness. The SRTR was selected for its large, nationally representative sample and its rich clinical detail, enabling a robust assessment of both short‐ and long‐term outcomes following LT.

### 2.2 Consent and Reporting

The requirement for informed consent was waived by the institutional review board due to the observational nature of the study and the use of de‐identified data (research protocol number PRO:13060220). All research activities were conducted in accordance with the ethical principles outlined in the Declarations of Helsinki and Istanbul [27]. Study findings are reported in compliance with the Strengthening the Reporting of Observational Studies in Epidemiology (STROBE) guidelines [28].

### 2.3 Inclusion and Exclusion Criteria

The primary objective of our analysis was to identify thresholds of donor–recipient age difference associated with increased risk of post‐transplant death or graft loss. To assess the effect of age difference regardless of graft type or donor category, we included recipients of livers from both living donors and donors after circulatory death (DCD). The cohort comprised pediatric and adult patients who underwent first‐time, single‐organ LT in the U.S. between January 1, 2011, and August 31, 2021, with follow‐up through December 31, 2024. Patients with a history of prior solid‐organ transplants or multivisceral grafts were excluded.

### 2.4 Variables

Recipient, donor, graft, and time‐dependent variables were selected based on established associations with post‐transplant outcomes.

Recipient variables included age at transplantation, sex, blood type, body mass index (BMI), serum total bilirubin, serum creatinine, international normalized ratio (INR), and serum sodium. These laboratory values were used to calculate the Model for End‐Stage Liver Disease Sodium (Na‐MELD) score, excluding exception points [29].

Additional recipient factors included history of diabetes, history of dialysis, primary indication for LT, and functional status, measured using the Karnofsky Performance Scale (KPS) [30], which ranges from 0 to 100, with higher values indicating better functional capacity.

Donor variables included age, sex, cause of death, and donor type: LD, DCD or donation after brain death (DBD).

Graft variables included graft type: whole or partial liver.

Time‐dependent variables included cold ischemia time (CIT), defined as the interval between aortic cross‐clamp and removal from cold preservation, and warm ischemia time (WIT), defined as the interval from the end of CIT to graft reperfusion.

Data on machine perfusion (normothermic or hypothermic) were not available in the registry and therefore could not be assessed. Nevetherless, published reports suggest that perfusion use remained relatively uncommon for most of the study period, with modest increase in the later years, particularly among older donors.

### 2.5 Stratification of the Study Population by Age

Recipients and donors were stratified by age at the time of transplantation. Recipients aged ≤18 years were classified as pediatric. Those aged 18.1–30 years were categorized as young adults. Individuals older than 30 years were grouped into five‐year age intervals. Donors and recipients ≥70 years old, were combined into a single category due to limited sample size.

### 2.6 Study Endpoints

The primary endpoints were:

1. To evaluate national trends in donor and recipient age over the study period.
2. To assess unadjusted five‐year patient survival, graft survival, and death‐censored graft survival across donor–recipient age groups.

The secondary endpoints were:

1. To estimate adjusted hazard ratios (aHRs) for mortality, graft failure, and death‐censored graft failure. 4. To evaluate potential interactions between donor and recipient age with respect to post‐transplant outcomes.

### 2.7 Statistical Analysis

The normality of continuous variables was assessed using the Shapiro–Wilk test. Normally distributed variables were summarized as means with standard deviations (SD); non‐normally distributed variables as medians with interquartile ranges (IQR). Group comparisons were performed using analysis of variance (ANOVA) or the Kruskal–Wallis test for continuous variables, and the χ^2^ test for categorical variables.

Unadjusted survival outcomes were analyzed using Kaplan–Meier methods, with group differences assessed by log‐rank tests. Patient survival was defined as time from transplantation to death or last follow‐up; graft survival as time from transplantation to death, relisting, or retransplantation; and death‐censored graft survival as time to graft failure, with deaths in patients with functioning grafts treated as censored events. Survival analyses were censored on December 31, 2024.

Associations between donor–recipient age and posttransplant outcomes were evaluated using Cox proportional hazards models. Models adjusted for recipient variables (sex, BMI, blood type, race/ethnicity, diabetes, dialysis, transplant indication, functional status, MELD‐Na); donor variables (age, sex, cause of death, race/ethnicity, BMI, donation type [DBD, DCD, LDLT]); graft type (whole vs. split); transplant year; and ischemia times (cold and warm). For each donor–recipient age category, the reference group comprised recipients who received grafts from donors at or below the median age for that group.

Collinearity was assessed using variance inflation factors. The proportional hazards assumption was evaluated using Schoenfeld residuals and Kaplan–Meier plots. Bonferroni correction was applied for multiple comparisons to control type I error.

A sensitivity analysis was conducted by excluding (a) recipients of living‐donor liver transplants and (b) recipients of grafts from DCD.

Missing covariate values were imputed using medians, given their robustness to outliers and suitability for skewed distributions. This single imputation approach might underestimate variance and did not account for the uncertainty inherent in missing data. While multiple imputation could reduce bias, the proportion of missingness was low (Supplementary Table 2) and the consistency of our primary sensitivity analysis suggested that any residual bias was unlikely to alter the effect estimates.

All statistical tests were two‐sided, with *P* < 0.05 considered statistically significant. Analyses were performed using SAS version 9.4 (SAS Institute Inc) and SPSS version 29.0.2.0 (IBM Corp).

## 3 Results

**FIGURE 1:**
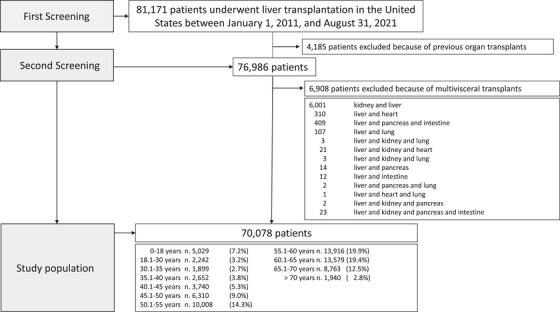
Flowchart detailing the study population selection and inclusion‐exclusion criteria.

How the study population was selected is detailed in the flowchart illustrated in Figure 1, while the baseline characteristics of the study population are summarized in Table 1. The median age of LT recipients was 57 years (interquartile range [IQR], 47–63), and 64.4% were male. The median MELD‐Na score at the time of transplantation was 19 (IQR, 13–29). Diabetes was present in 25.1% of recipients, and 10.1% required dialysis within the week prior to LT. The most common indications for transplantation were alcohol‐associated liver disease (ALD, 22.8%), metabolic‐associated fatty liver disease (MAFLD, 20.8%), hepatocellular carcinoma (HCC, 19.3%), and hepatitis C virus‐related cirrhosis (HCV, 10.8%).

**TABLE 1:** Sociodemographic and clinical characteristics of the study population.

Characteristics, *n* (%)	Recipients, *n* 70 078
**Age, years, median (IQR)**	57 (47.0–63.0)
**Sex, male** *n* **(%)**	45 113 (64.4)
**Blood group,** *n* **(%)**	
A	25 698 (36.7)
AB	3346 (4.8)
B	9358 (13.4)
O	31 676 (45.2)
**Body mass index,** *n* **(%)**	
< 18.5	5529 (7.9)
18.5–25	18 012 (25.7)
25.1–30	21 996 (31.4)
30.1–40	21 551 (30.8)
>40	2990 (4.3)
**MELD‐Na score without exceptional points, median (IQR)**	19 (13–29)
**Mean performance status**–**Karnofsky**–**mean (SD)**	50 (23)
**History of diabetes,** *n* **(%)**	17 575 (25.1)
**Need for dialysis before transplantation,** *n* **(%)**	7084 (10.1)
**Primary indication for liver transplant,** *n* **(%)**	
Alcoholic liver disease	15 950 (22.8)
Viral hepatitis B	897 (1.3)
Viral hepatitis C	7550 (10.8)
Hepatocellular carcinoma	13 499 (19.3)
Metabolic associated fatty liver disease (MAFLD)	14 540 (20.8)
Primary biliary cirrhosis	1848 (2.6)
Primary sclerosing cholangitis	1651 (2.4)
Autoimmune diseases	2313 (3.3)
Other	11 830 (16.9)
**Donor age, years, median (IQR)**	40.0 (26–53)
**Donor sex, male,** *n* **(%)**	41 662 (59.5)
**Cold ischemia time, hours, median (IQR)**	5.73 (4.43–7.18)
**Warm ischemia time, minutes, median (IQR)**	37 (29.0–47.0)
**Living donor,** *n* **(%)**	3829 (5.5)
**Donation after cardiocirculatory arrest,** *n* **(%)**	4805 (7.3)
**Donor's primary cause of death,** *n* **(%)**	
Anoxic event	25 152 (35.9)
Cerebrovascular accident	19 888 (28.4)
Other or unknown	25 038 (35.72)
**Partial liver graft,** *n* **(%)**	5733 (8.1)
**Whole liver graft,** *n* **(%)**	64 345 (91.9)

*Note:* Interquartile ranges (IQR); Metabolic Associated Fatty Liver Disease (MAFLD).

The mean donor age was 40 years (IQR 26–53), and 59.5% of donors were male. Organs from DBD represented 87.2% of the total number of grafts, LDLTs were performed in 5.5% of recipients and 7.3% of the grafts were from DCD donors. Whole liver grafts accounted for 91.9% of cases, whereas 8.1% of LTs were performed using split grafts. The median CIT was 5.7 h (IQR, 4.4–7.1), and the median WIT was 37 min (IQR, 29.0–47.0). The distribution of donor age across recipient age categories is shown in Supplementary Table 1. The extent of the missing data is detailed in Supplementary Table 2.

### 3.1 Donor and Recipient Age Trends

**FIGURE 2:**
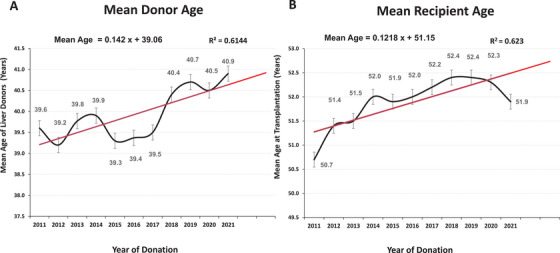
Trends in donor and recipient age over time in the United States.

**FIGURE 3:**
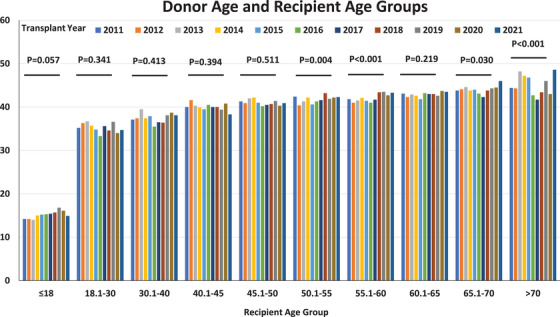
Trends in donor age by recipient age group in the United States.

**FIGURE 4:**
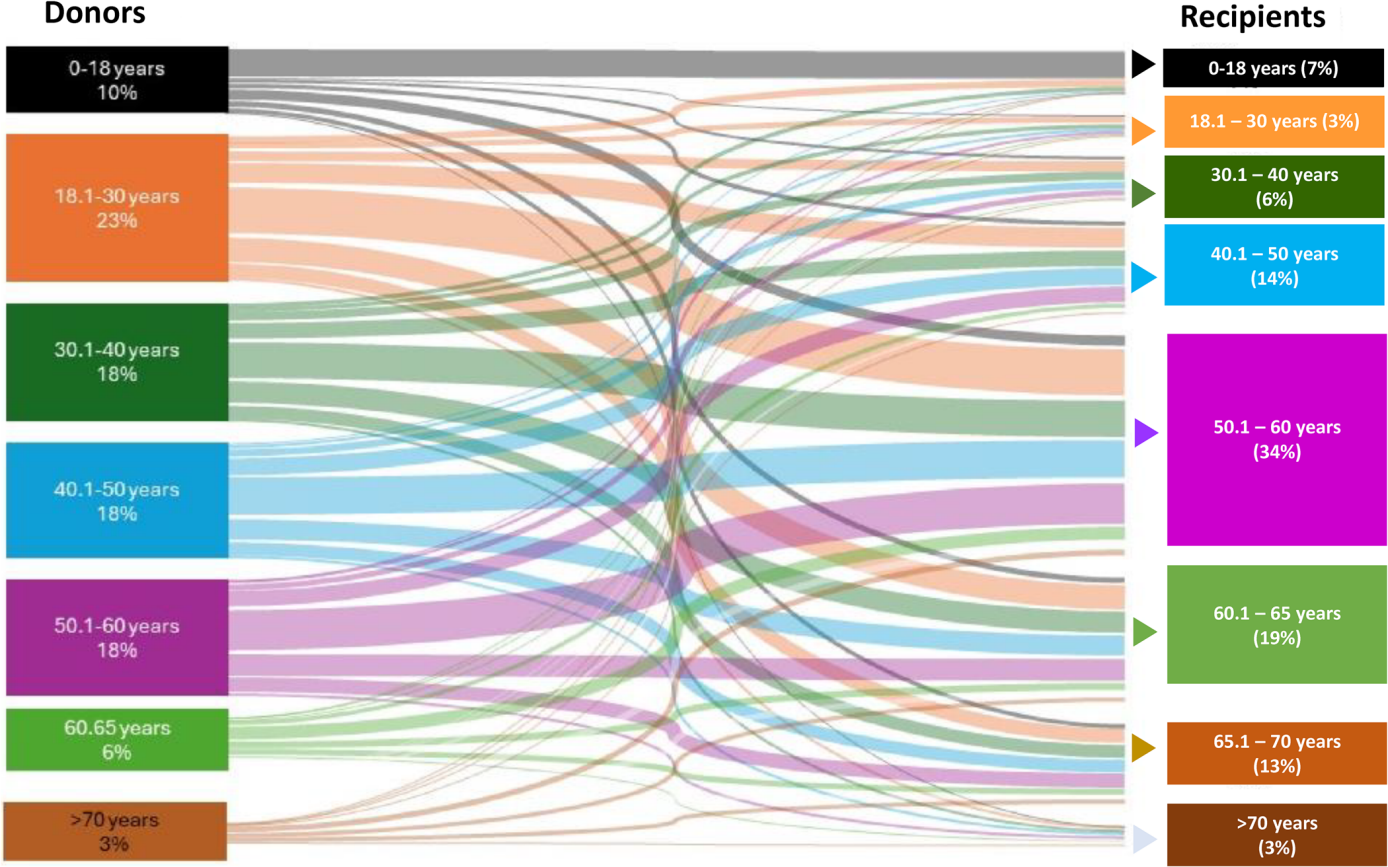
Donor‐recipient age allocation patterns. Sankey diagram depicting organ allocation flows across donor and recipient age categories. Wider bands represent greater transplant volume.

During the study period, the mean donor age increased from 39.6 years (SD, 17.8) in 2011 to 40.9 years (SD, 16.6) in 2021 (*p* = 0.004; Figure 2A). Similarly, the mean recipient age rose from 50.7 years (SD, 16.7) to 51.9 years (SD, 15.9) over the same period (*p* = 0.003; Figure 2B). Over the study period, recipients belonging to older age groups experienced an increase in the age of their corresponding donors (Figure 3). Organ allocation patterns across donor‐recipient age categories are depicted in the Sankey diagram in Figure 4.

### 3.2 Recipient Survival

Unadjusted five‐year patient survival declined with increasing donor–recipient age disparity, particularly among pediatric recipients (92% vs. 75%; *P* for trend = 0.02) and adults aged 35.1–40 years (96% vs. 73%; *P* for trend < 0.001), with minimal differences in recipients older than 65 years (Figure 5A).

FIGURE 5Donor age and 5‐year patient survival rate by recipient age. (A) Unadjusted 5‐year outcomes stratified by donor–recipient age combinations. (B) Radar plot illustrating donor age thresholds associated with significantly increased adjusted hazard ratios (aHRs) for patient mortality across recipient age groups. Reference groups include recipients of grafts from donors at or below the median donor age for each recipient age category.

**Figure:**
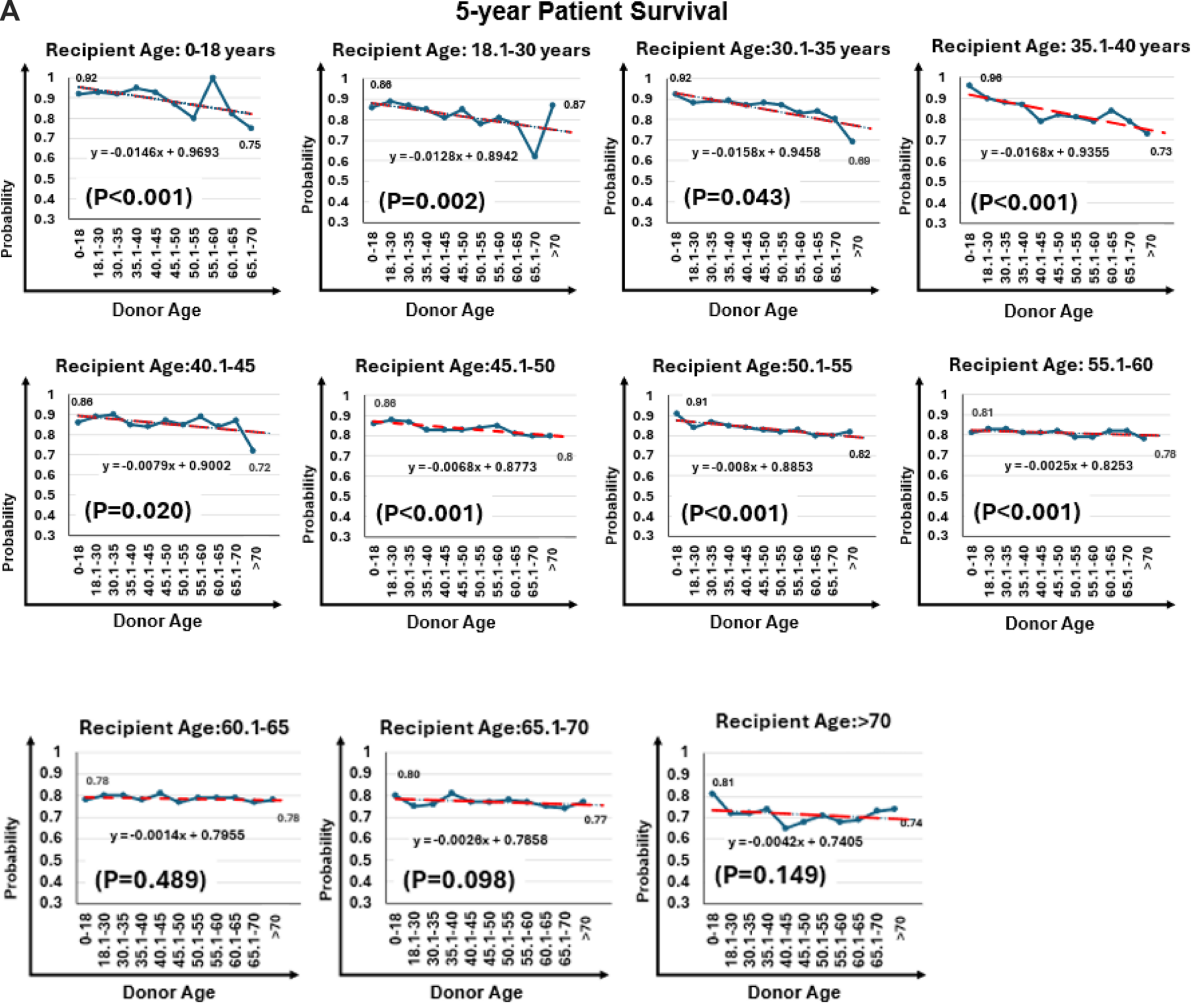

**Figure:**
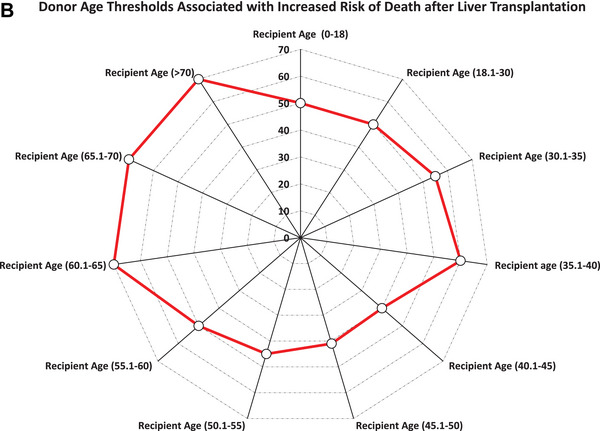

In adjusted analyses (Table 2), donor age >50 years was associated with increased mortality in pediatric recipients (aHR, 6.48; 95% CI, 1.92–21.83; *p* = 0.003) and in adults aged 18.1–30 years with donors aged 50.1–60 years (aHRs, 2.79–2.82; *p* < 0.05). Among recipients aged 30.1–40 years, increased mortality was observed only with donors ≥55 years (aHRs, 2.57–3.08; *p* < 0.05).

**TABLE 2:** Adjusted hazard ratios (aHRs) for posttransplant mortality, stratified by donor–recipient age groups. Recipients of grafts from donors at or below the median donor age served as the reference group for all comparisons.

Recipient age (years)	Donor age (years)	*n* patients	aHR	95% LCI	95% UCI	*p* value
≤ 18 (Median donor age:14)	≤14	2631	**Reference**			
	14.1–18	696	0.999	0.405	2.464	0.998
	18.1–30	919	0.927	0.371	2.317	0.871
	30.1–35	295	1.046	0.270	4.051	0.949
	35.1–40	197	1.533	0.368	6.395	0.557
	40.1–45	123	0.672	0.121	3.740	0.650
	45.1–50	81	1.911	0.320	11.393	0.477
	**>50**	**77**	**6.478**	**1.922**	**21.831**	**0.003**
18.1–30 (Median donor age:33)	≤33	1190	**Reference**			
	33.1–35	92	2.407	0.865	6.696	0.092
	35.1–40	203	1.532	0.712	3.297	0.275
	40.1–45	210	1.571	0.703	3.511	0.271
	45.1–50	199	0.794	0.333	1.889	0.601
	**50.1–55**	**173**	**2.785**	**1.320**	**5.875**	**0.007**
	**55.1–60**	**117**	**2.822**	**1.253**	**6.36**	**0.012**
	>60	102	2.625	0.866	7.950	0.088
30.1–35 (Median donor age:36)	≤36	1039	**Reference**			
	36.1–40	119	1.187	0.340	4.148	0.788
	40.1–45	143	0.396	0.121	1.293	0.125
	45.1–50	179	0.792	0.295	2.129	0.644
	50.1–55	172	0.798	0.285	2.233	0.667
	**55.1–60**	**122**	**3.081**	**1.057**	**8.977**	**0.039**
	>60	114	3.134	0.828	11.862	0.092
35.1–40 (Median donor age:38)	≤38	1405	**Reference**			
	38.1–40	65	1.320	0.281	6.205	0.725
	40.1–45	264	1.615	0.761	3.426	0.212
	45.1–50	287	1.652	0.809	3.374	0.168
	50.1–55	241	1.974	0.926	4.208	0.078
	55.1–60	185	1.560	0.672	3.620	0.301
	**>60**	**205**	**2.574**	**1.075**	**6.167**	**0.034**
40.1–45 (Median donor age:40)	≤40	1936	**Reference**			
	**40.1–45**	**365**	**2.04**	**1.179**	**3.529**	**0.011**
	45.1–50	404	0.740	0.393	1.395	0.352
	**50.1–55**	**344**	**2.161**	**1.288**	**3.626**	**0.004**
	55.1–60	312	1.395	0.755	2.575	0.288
	60.1–65	220	1.417	0.676	2.971	0.356
	**>65**	**159**	**3.980**	**1.747**	**9.068**	**0.001**
45.1–50 (Median donor age:41)	≤41	3217	**Reference**			
	**41.1–45**	**465**	**1.636**	**1.109**	**2.413**	**0.013**
	**45.1–50**	**710**	**1.387**	**0.989**	**1.944**	**0.058**
	**50.1–55**	**616**	**1.483**	**1.045**	**2.106**	**0.027**
	**55.1–60**	**583**	**1.510**	**1.036**	**2.201**	**0.032**
	**60.1–65**	**389**	**1.640**	**1.079**	**2.493**	**0.021**
	**65.1–70**	**210**	**2.175**	**1.293**	**3.658**	**0.003**
	>70	120	1.635	0.806	3.319	0.173
50.1–55 (Median donor age:42)	≤42	4912	**Reference**			
	42.1–45	723	1.061	0.783	1.439	0.701
	**45.1–50**	**1041**	**1.454**	**1.152**	**1.834**	**0.002**
	**50.1–55**	**1145**	**1.310**	**1.039**	**1.651**	**0.022**
	**55.1–60**	**940**	**1.473**	**1.122**	**1.933**	**0.005**
	**60.1–65**	**596**	**1.955**	**1.459**	**2.619**	**<0.001**
	**65.1–70**	**377**	**1.576**	**1.067**	**2.326**	**0.022**
	**>70**	**274**	**1.849**	**1.212**	**2.820**	**0.004**
55.1–60 (Median donor age:42)	≤42	7010	**Reference**			
	42.1–45	734	1.044	0.781	1.396	0.770
	45.1–50	1421	1.094	0.891	1.343	0.390
	**50.1–55**	**1531**	**1.293**	**1.068**	**1.567**	**0.008**
	**55.1–60**	**1281**	**1.598**	**1.312**	**1.948**	**<0.001**
	60.1–65	957	1.097	0.856	1.404	0.465
	65.1–70	550	1.228	0.899	1.677	0.198
	**>70**	**432**	**1.539**	**1.134**	**2.090**	**0.006**
60.1–65 (Median donor age:43)	≤43	6938	**Reference**			
	43.1–45	500	0.865	0.613	1.219	0.406
	45.1–50	1306	1.147	0.928	1.418	0.205
	50.1–55	1384	1.005	0.814	1.242	0.962
	55.1–60	1280	0.998	0.799	1.245	0.983
	60.1–65	953	1.233	0.978	1.553	0.076
	65.1–70	655	1.061	0.793	1.419	0.692
	**>70**	**554**	**1.385**	**1.014**	**1.892**	**0.041**
65.1–70 (Median donor age:44)	≤44	4396	**Reference**			
	44.1–45	159	1.349	0.774	2.351	0.291
	45.1–50	850	1.152	0.877	1.513	0.309
	50.1–55	896	0.911	0.677	1.225	0.536
	55.1–60	808	0.969	0.719	1.306	0.835
	60.1–65	681	0.907	0.654	1.256	0.556
	65.1–70	465	1.039	0.730	1.481	0.830
	>70	494	1.262	0.897	1.776	0.181
>70 (Median donor age:46)	≤46	1002	**Reference**			
	46.1–50	122	1.349	0.585	3.110	0.482
	50.1–55	212	0.730	0.392	1.358	0.321
	55.1–60	198	1.553	0.857	2.816	0.147
	60.1–65	147	1.250	0.612	2.551	0.399
	65.1–70	125	1.350	0.672	2.715	0.399
	>70	134	1.781	0.888	3.571	0.104

*Note:* Adjusted hazard ratios (aHRs) were estimated using multivariable Cox regression models accounting for recipient characteristics (sex, BMI, blood type, race/ethnicity, diabetes history, dialysis history, primary indication for LT, functional status, and MELD‐Na score); donor characteristics (sex, race/ethnicity, primary cause of death, BMI, and type of donation—DBD, DCD, or LDLT); organ characteristics (whole vs. split graft); and transplant‐related factors, including year of transplantation, warm ischemia time (WIT), and cold ischemia time (CIT).

Bold values are those that resulted statistically significant *p* value <0.05.

From ages 40.1 to 60 years, mortality risk increased progressively with donor age. Significant associations were observed for donors aged ≥40 years in recipients aged 40.1–45 years (aHR, 2.04; 95% CI, 1.18–3.53; *p* = 0.011) and for donors >60 years in those aged 50.1–55 years (aHR, 1.95; 95% CI, 1.46–2.62; *p* < 0.001). Among recipients aged 55.1–60 years, risk remained elevated with donor age ≥50 years (aHRs up to 1.60; *p* < 0.001).

In recipients aged 60.1–65 years, only donors >70 years were associated with higher mortality (aHR, 1.39; 95% CI, 1.01–1.89; *p* = 0.041). No significant associations were observed among recipients older than 65 years (*p* > 0.18 for all). Donor age thresholds associated with increased adjusted mortality across recipient age groups are shown in Figure 5B.

#### 3.2.1 Graft Survival

Unadjusted five‐year graft survival declined with increasing donor–recipient age disparity, particularly among pediatric recipients (85% vs. 50%) and adults aged 35.1–40 years (93% vs. 50%) (*P* for trend < 0.001 for both; Figure 6A). In contrast, survival was relatively stable across donor age groups among recipients aged 65.1–70 years (79% vs. 73%; *p* = 0.10) and those older than 70 years (79% vs. 72%; *p* = 0.11).

FIGURE 6Donor age and 5‐year graft survival rate by recipient age. (A) Unadjusted 5‐year graft survival stratified by donor–recipient age combinations. (B) Radar plot illustrating donor age thresholds associated with significantly increased adjusted hazard ratios (aHRs) for graft loss across recipient age groups. Reference groups include recipients of grafts from donors at or below the median donor age for each recipient age category.

**Figure:**
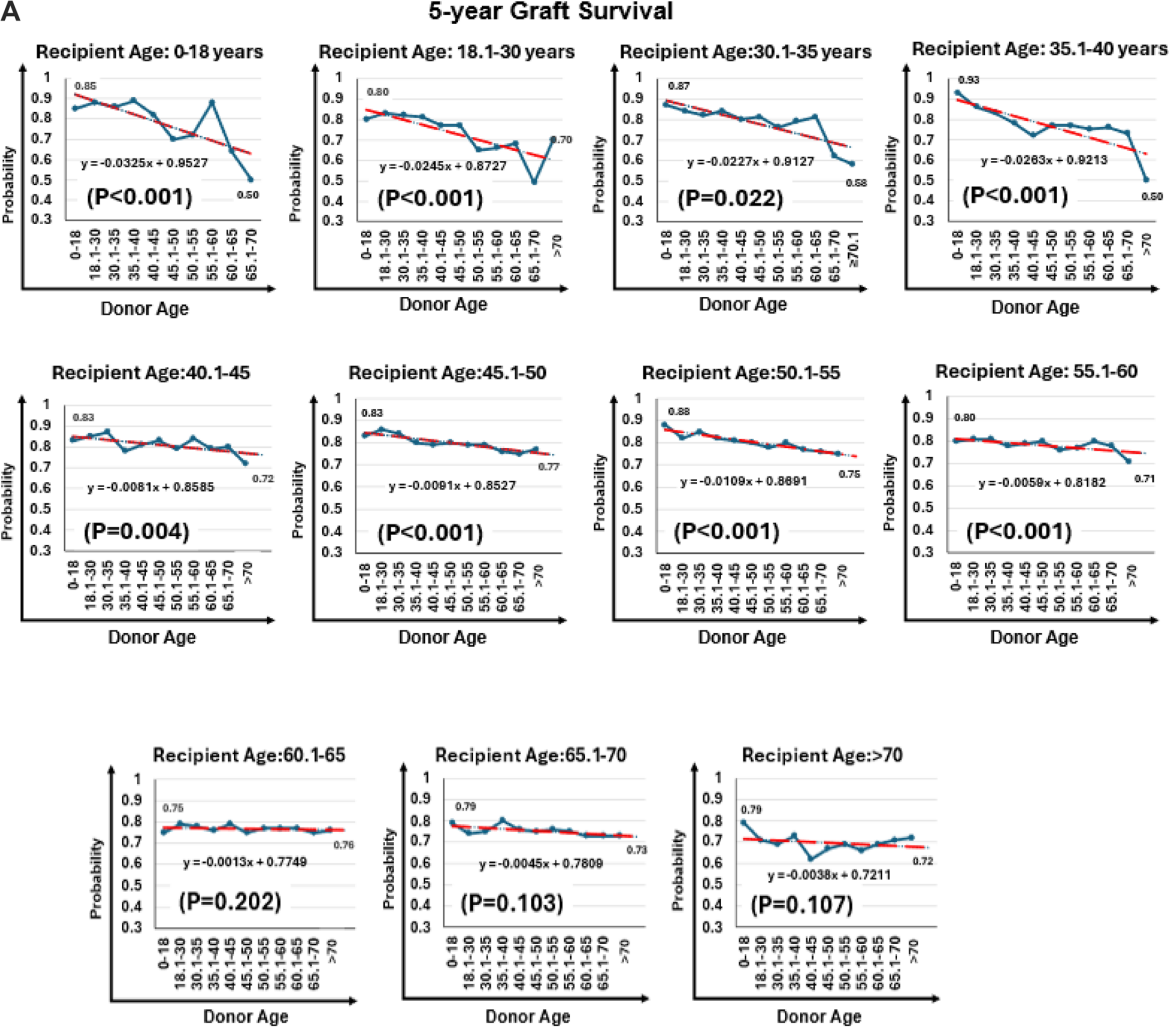

**Figure:**
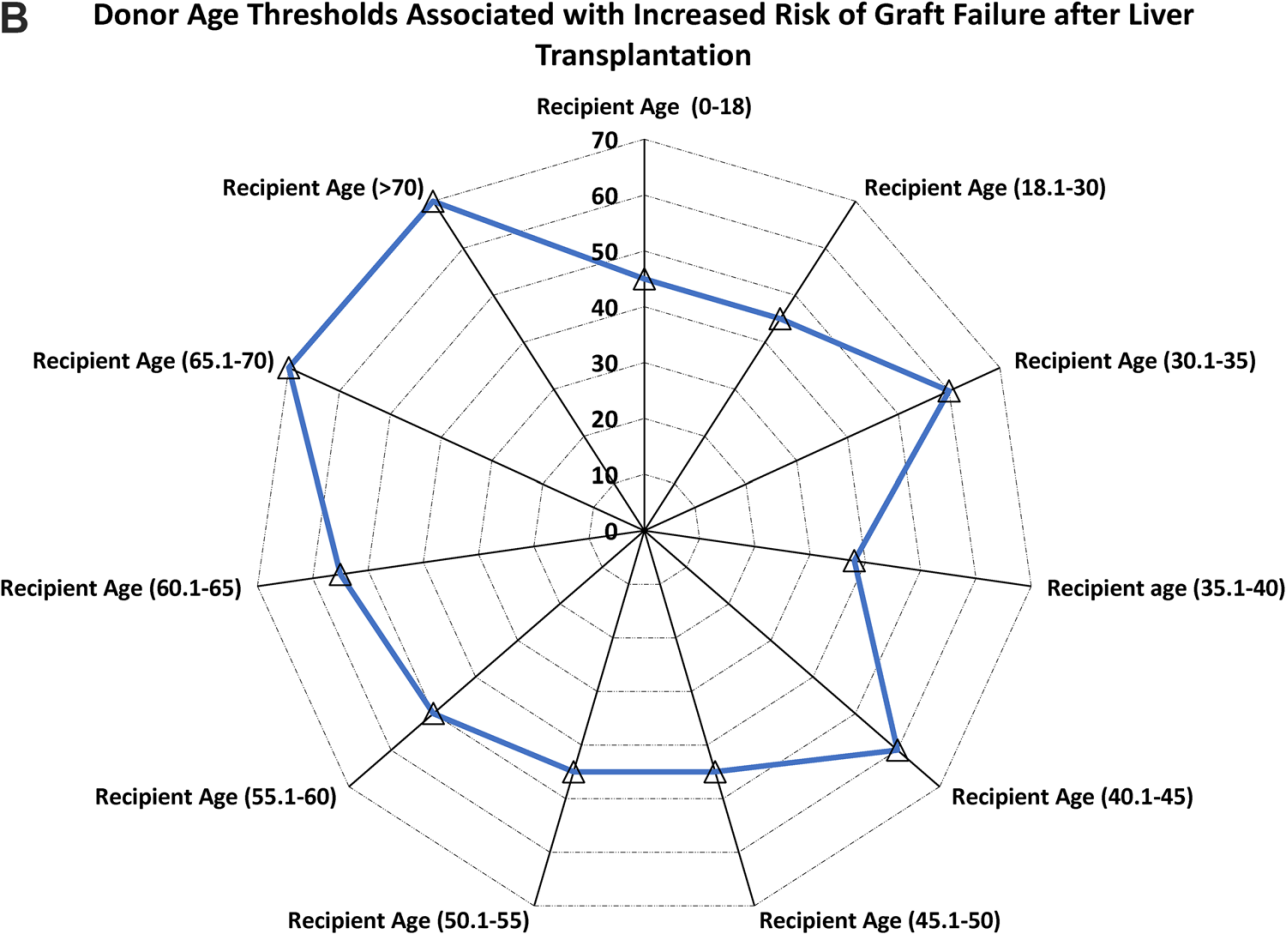

In adjusted analyses (Table 3), older donor age was associated with increased graft loss in recipients younger than 60 years, most notably in pediatric recipients (aHR, 4.98; 95% CI, 1.45–17.08) and those aged 18.1–30 years (aHR, 6.17; 95% CI, 2.58–14.77 for donors aged 55.1–60 years). Risk increased progressively with donor age among recipients aged 40.1–60 years, peaking with donors aged 60.1–65 years (aHR, 3.08; 95% CI, 2.03–4.68). In recipients aged 60.1–65 years, elevated risk persisted (aHR, 2.53; 95% CI, 1.68–3.82), but no significant associations were observed in those older than 65 years (*p* > 0.17 for all).

**TABLE 3:** Adjusted hazard ratios (aHRs) for graft failure after LT, stratified by donor–recipient age groups. Recipients of grafts from donors at or below the median donor age served as the reference group for all comparisons.

Recipient age (years)	Donor age (years)	*n* patients	aHR	95% LCI	95% UCI	*p* value
≤18 (Median donor age: 14)	≤14	2631	**Reference**			
	14.1–18	696	1.114	0.409	3.033	0.833
	18.1–30	919	0.794	0.274	2.300	0.671
	30.1–35	295	1.581	0.450	5.558	0.475
	35.1–40	197	3.444	0.935	12.687	0.063
	40.1–45	123	3.136	0.841	11.690	0.089
	**45.1–50**	**81**	**50.830**	**15.993**	**161.555**	**<0.001**
	**>50**	**77**	**4.981**	**1.453**	**17.084**	**0.011**
18.1–30 (Median donor age: 33)	≤33	1190	**Reference**			
	33.1–35	92	2.542	0.757	8.540	0.131
	35.1–40	203	0.991	0.369	2.659	0.986
	40.1–45	210	1.329	0.485	3.641	0.581
	**45.1–50**	**199**	**2.381**	**1.052**	**5.388**	**0.037**
	**50.1–55**	**173**	**4.930**	**2.027**	**11.988**	**<0.001**
	**55.1–60**	**117**	**6.169**	**2.577**	**14.768**	**<0.001**
	**>60**	**102**	**3.387**	**1.240**	**9.253**	**0.017**
30.1–35 (Median donor age: 36)	≤36	1039	**Reference**			
	36.1–40	119	0.970	0.293	3.219	0.961
	40.1–45	143	0.985	0.357	2.711	0.976
	45.1–50	179	0.998	0.372	2.682	0.997
	50.1–55	172	1.006	0.345	2.934	0.992
	55.1–60	122	1.029	0.239	4.425	0.969
	>60	114	1.078	0.293	3.965	0.910
35.1–40 (Median donor age: 38)	≤38	1405	**Reference**			
	**38.1–40**	**65**	**5.637**	**2.054**	**15.470**	**<0.001**
	40.1–45	264	1.063	0.340	3.323	0.917
	45.1–50	287	1.254	0.474	3.316	0.648
	50.1–55	241	1.091	0.393	3.029	0.867
	55.1–60	185	1.090	0.351	3.379	0.882
	>60	205	0.527	1.387	0.504	3.821
40.1–45 (Median donor age: 40)	≤40	1936	**Reference**			
	40.1–45	365	1.763	0.772	4.022	0.178
	45.1–50	404	0.832	0.337	2.054	0.690
	50.1–55	344	1.493	0.674	3.307	0.324
	55.1–60	312	1.789	0.791	4.045	0.162
	**60.1–65**	**220**	**2.778**	**1.170**	**6.597**	**0.021**
	**>65**	**159**	**3.139**	**1.400**	**7.039**	**0.005**
45.1–50 (Median donor age: 41)	≤41	3217	**Reference**			
	**41.1–45**	465	1.293	0.655	2.552	0.458
	**45.1–50**	**710**	**2.069**	**1.235**	**3.466**	**0.006**
	**50.1–55**	**616**	**2.263**	**1.360**	**3.767**	**0.002**
	**55.1–60**	583	1.486	0.800	2.758	0.210
	**60.1–65**	**389**	**2.650**	**1.481**	**4.740**	**0.001**
	**65.1–70**	**210**	**3.025**	**1.536**	**5.958**	**0.001**
	>70	120	2.107	0.859	5.166	0.103
50.1–55 (Median donor age: 42)	≤42	4912	**Reference**			
	42.1–45	723	1.633	0.956	2.789	0.072
	**45.1–50**	**1041**	**1.797**	**1.205**	**2.678**	**0.004**
	**50.1–55**	**1145**	**1.514**	**1.020**	**2.248**	**0.040**
	**55.1–60**	**940**	**1.878**	**1.220**	**2.890**	**0.004**
	**60.1–65**	**596**	**3.082**	**2.031**	**4.675**	**<0.001**
	**65.1–70**	**377**	**3.000**	**1.791**	**5.027**	**0.001**
	>70	274	1.812	0.894	3.675	0.099
55.1–60 (Median donor age: 42)	≤42	7010	**Reference**			
	42.1–45	734	0.866	0.484	1.549	0.627
	45.1–50	1421	1.139	0.772	1.681	0.511
	**50.1–55**	**1531**	**1.688**	**1.208**	**2.358**	**0.002**
	**55.1–60**	**1281**	**1.817**	**1.290**	**2.559**	**<0.001**
	60.1–65	957	1.424	0.931	2.178	0.103
	65.1–70	550	1.578	0.936	2.659	0.087
	**>70**	**432**	**2.194**	**1.337**	**3.599**	**0.002**
60.1–65 (Median donor age: 43)	≤43	6938	**Reference**			
	43.1–45	500	1.001	0.487	2.058	0.997
	45.1–50	1306	0.780	0.455	1.335	0.364
	50.1–55	1384	1.025	0.645	1.628	0.918
	**55.1–60**	**1280**	**1.660**	**1.096**	**2.514**	**0.017**
	**60.1–65**	**953**	**2.534**	**1.680**	**3.822**	**<0.001**
	65.1–70	655	1.279	0.711	2.299	0.411
	>70	554	0.983	0.459	2.101	0.964
65.1–70 (Median donor age: 44)	≤44	4396	**Reference**			
	44.1–45	159	1.667	0.443	6.269	0.449
	45.1–50	850	1.681	0.929	3.041	0.086
	50.1–55	896	1.164	0.590	2.296	0.661
	55.1–60	808	1.029	0.523	2.025	0.935
	60.1–65	681	0.812	0.334	1.977	0.647
	65.1–70	465	1.272	0.569	2.846	0.558
	>70	494	1.670	0.797	3.501	0.174
>70 (Median donor age: 46)	≤46	1002	**Reference**			
	46.1–50	122	0.185	0.003	10.437	0.412
	50.1–55	212	0.961	0.215	4.289	0.959
	55.1–60	198	0.219	0.018	2.639	0.231
	60.1–65	147	0.261	0.016	4.345	0.349
	65.1–70	125	1.138	0.180	7.176	0.891
	>70	134	0.646	0.086	4.879	0.672

*Note:* Adjusted hazard ratios (aHRs) were estimated using multivariable Cox regression models that accounted for recipient characteristics (sex, BMI, blood type, race/ethnicity, history of diabetes, history of dialysis, primary indication for LT, functional status, and MELD‐Na score); donor characteristics (sex, race/ethnicity, primary cause of death, BMI, and type of organ donation—DBD, DCD, or LDLT); organ characteristics (whole vs. split graft); and transplant‐related factors, including year of transplantation, warm ischemia time (WIT), and cold ischemia time (CIT).

Bold values are those that resulted statistically significant *p* value <0.05.

Donor age thresholds associated with increased graft loss by recipient age group are shown in Figure 6B.

#### 3.2.2 Death‐Censored Graft Survival

Unadjusted five‐year death‐censored graft survival declined with increasing donor age among pediatric (85% vs. 71%; *P* for trend = 0.002) and younger adult recipients aged 35.1 to 40 years (92% vs. 61%; *P* for trend < 0.001), but remained stable in older recipients (65.1–70 years: 91% vs. 88%, *p* = 0.01; >70 years: 94% vs. 93%, *p* = 0.29) (Figure 7A).

FIGURE 7Donor age and 5‐year death‐censored graft survival rate by recipient age. (A) Unadjusted 5‐year death‐censored graft survival stratified by donor–recipient age combinations. (B) Radar plot illustrating donor age thresholds associated with significantly increased adjusted hazard ratios (aHRs) for death‐censored graft loss across recipient age groups. Reference groups include recipients of grafts from donors at or below the median donor age for each recipient age category.

**Figure:**
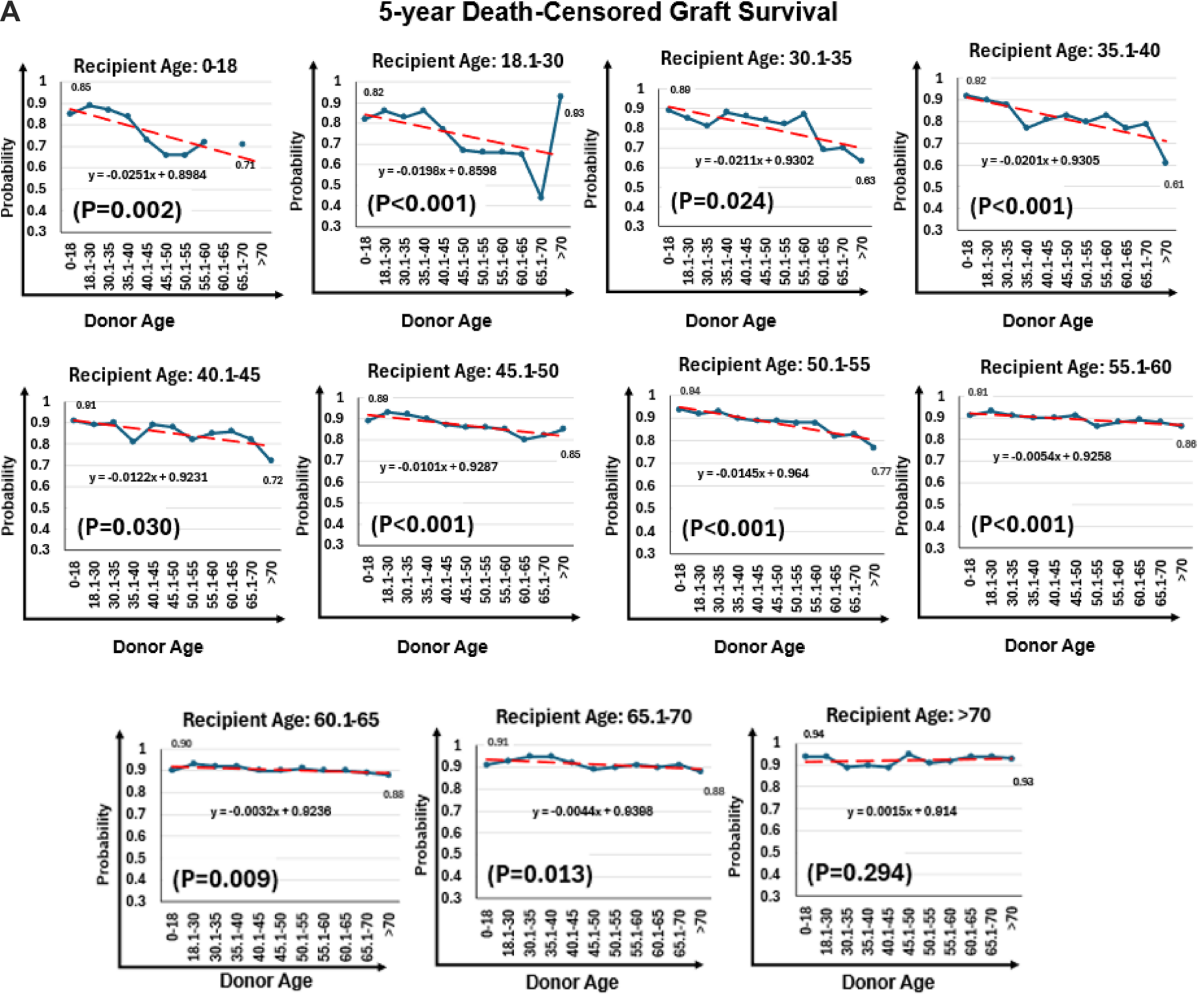

**Figure:**
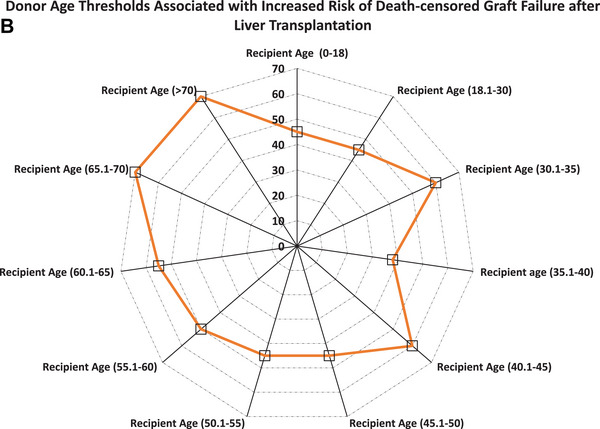

In adjusted analyses (Table 4), donor age >45 years was associated with markedly increased risk of graft failure in pediatric recipients (aHR, 55.9; 95% CI, 17.1–183.2; *p* < 0.001) and significantly elevated risk among adults aged 18.1–30 years, particularly for donors aged 55.1–60 years (aHR, 5.75; 95% CI, 2.42–13.66; *p* < 0.001). In recipients aged 45.1–60 years, older donor age remained significantly associated with risk, especially for donors aged 60.1–70 years (aHRs ∼2.96–2.97; *p* < 0.001). No consistent association was observed among recipients ≥65 years. Adjusted age‐risk thresholds are shown in Figure 7B.

**TABLE 4:** Adjusted hazard ratios (aHRs) for death‐censored graft loss, stratified by donor–recipient age groups. Recipients of grafts from donors at or below the median donor age served as the reference group for all comparison.

Recipient age (years)	Donor age (years)	*n* patients	aHR	95% LCI	95% UCI	*p* value
≤18 (Median donor age: 14)	≤14	2631	**Reference**			
	14.1–18	696	1.114	0.418	2.968	0.829
	18.1–30	919	0.780	0.273	2.224	0.642
	30.1–35	295	1.534	0.437	5.393	0.504
	35.1–40	197	3.359	0.917	12.299	0.067
	40.1–45	123	3.041	0.842	10.992	0.090
	**45.1–50**	**81**	**55.905**	**17.062**	**183.176**	**<0.001**
	**>50**	**77**	**4.824**	**1.432**	**16.248**	**0.011**
18.1–30 (Median donor age: 33)	≤33	1190	**Reference**			
	33.1–35	92	2.537	0.796	8.086	0.116
	35.1–40	203	1.039	0.389	2.775	0.939
	40.1–45	210	1.294	0.483	3.468	0.609
	**45.1–50**	**199**	**2.562**	**1.142**	**5.748**	**0.023**
	**50.1–55**	**173**	**4.845**	**2.012**	**11.668**	**<0.001**
	**55.1–60**	**117**	**5.746**	**2.416**	**13.664**	**<0.001**
	**>60**	**102**	**3.122**	**1.180**	**8.260**	**0.022**
30.1–35 (Median donor age: 36)	≤36	1039	**Reference**			
	36.1–40	119	0.974	0.291	3.259	0.966
	40.1–45	143	0.983	0.354	2.727	0.973
	45.1–50	179	0.997	0.370	2.683	0.995
	50.1–55	172	1.002	0.339	2.958	0.997
	55.1–60	122	1.026	0.239	4.398	0.972
	>60	114	1.074	0.293	3.940	0.914
35.1–40 (Median donor age: 38)	≤38	1405	**Reference**			
	**38.1–40**	**65**	**5.229**	**1.856**	**14.729**	**0.002**
	40.1–45	264	1.011	0.322	3.178	0.985
	45.1–50	287	1.250	0.472	3.310	0.653
	50.1–55	241	1.088	0.389	3.037	0.873
	55.1–60	185	1.111	0.357	3.457	0.856
	>60	205	1.332	0.479	3.699	0.583
40.1–45 (Median donor age: 40)	≤40	1936	**Reference**			
	40.1–45	365	1.662	0.733	3.771	0.224
	45.1–50	404	0.805	0.324	2.000	0.641
	50.1–55	344	1.416	0.643	3.118	0.388
	55.1–60	312	1.685	0.751	3.779	0.206
	**60.1–65**	**220**	**2.551**	**1.080**	**6.026**	**0.033**
	**>65**	**159**	**2.940**	**1.323**	**6.536**	**0.008**
45.1–50 (Median donor age: 41)	≤41	3217	**Reference**			
	**41.1–45**	465	1.304	0.648	2.623	0.457
	**45.1–50**	**710**	**1.970**	**1.181**	**3.287**	**0.009**
	**50.1–55**	**616**	**2.107**	**1.276**	**3.479**	**0.004**
	**55.1–60**	583	1.382	0.753	2.539	0.296
	**60.1–65**	**389**	**2.353**	**1.338**	**4.140**	**0.003**
	**65.1–70**	**210**	**2.583**	**1.335**	**4.995**	**0.005**
	>70	120	1.915	0.802	4.575	0.143
50.1–55 (Median donor age: 42)	≤42	4912	**Reference**			
	**42.1–45**	**723**	**1.616**	**0.944**	**2.766**	**0.080**
	**45.1–50**	**1041**	**1.783**	**1.195**	**2.661**	**0.005**
	50.1–55	1145	1.464	0.986	2.175	0.059
	**55.1–60**	**940**	**1.837**	**1.194**	**2.825**	**0.006**
	**60.1–65**	**596**	**2.973**	**1.967**	**4.493**	**<0.001**
	**65.1–70**	**377**	**2.963**	**1.772**	**4.953**	**<0.001**
	>70	274	1.807	0.890	3.671	0.102
55.1–60 (Median donor age: 42)	≤42	7010	**Reference**			
	42.1–45	734	0.867	0.484	1.553	0.632
	45.1–50	1421	1.143	0.775	1.686	0.501
	**50.1–55**	**1531**	**1.630**	**1.167**	**2.275**	**0.004**
	**55.1–60**	**1281**	**1.764**	**1.255**	**2.480**	**0.001**
	60.1–65	957	1.367	0.895	2.086	0.148
	65.1–70	550	1.491	0.889	2.501	0.130
	**>70**	**432**	**2.057**	**1.254**	**3.372**	**0.004**
60.1–65 (Median donor age: 43)	≤43	6938	**Reference**			
	43.1–45	500	0.997	0.485	2.050	0.993
	45.1–50	1306	0.720	0.423	1.225	0.226
	50.1–55	1384	0.958	0.612	1.501	0.852
	**55.1–60**	**1280**	**1.513**	**1.010**	**2.267**	**0.045**
	**60.1–65**	**953**	**2.236**	**1.510**	**3.312**	**<0.001**
	65.1–70	655	1.131	0.649	1.971	0.665
	>70	554	0.879	0.424	1.823	0.729
65.1–70 (Median donor age: 44)	≤44	4396	**Reference**			
	44.1–45	159	1.720	0.432	6.858	0.442
	45.1–50	850	1.685	0.927	3.065	0.087
	50.1–55	896	1.105	0.557	2.193	0.775
	55.1–60	808	0.978	0.496	1.929	0.949
	60.1–65	681	0.767	0.316	1.862	0.558
	65.1–70	465	1.224	0.551	2.718	0.620
	>70	494	1.761	0.846	3.664	0.130
>70 (Median donor age: 46)	≤46	1002	**Reference**			
	46.1–50	122	0.195	0.003	12.197	0.439
	50.1–55	212	0.760	0.171	3.373	0.718
	55.1–60	198	0.200	0.016	2.445	0.208
	60.1–65	147	0.278	0.023	3.398	0.316
	65.1–70	125	1.122	0.162	7.759	0.907
	>70	134	0.611	0.078	4.810	0.639

*Note:* Adjusted hazard ratios (aHRs) were estimated using multivariable Cox regression models adjusted for recipient characteristics (sex, BMI, blood type, race/ethnicity, history of diabetes, history of dialysis, primary indication for LT, functional status, and MELD‐Na score); donor characteristics (sex, race/ethnicity, primary cause of death, BMI, and type of organ donation—DBD, DCD, or LDLT); organ characteristics (whole vs. split graft); and transplant‐related factors, including year of transplantation, warm ischemia time (WIT), and cold ischemia time (CIT).

Bold values are those that resulted statistically significant *p* value <0.05.

#### 3.2.3 Donor–Recipient Age Interaction

**FIGURE 8:**
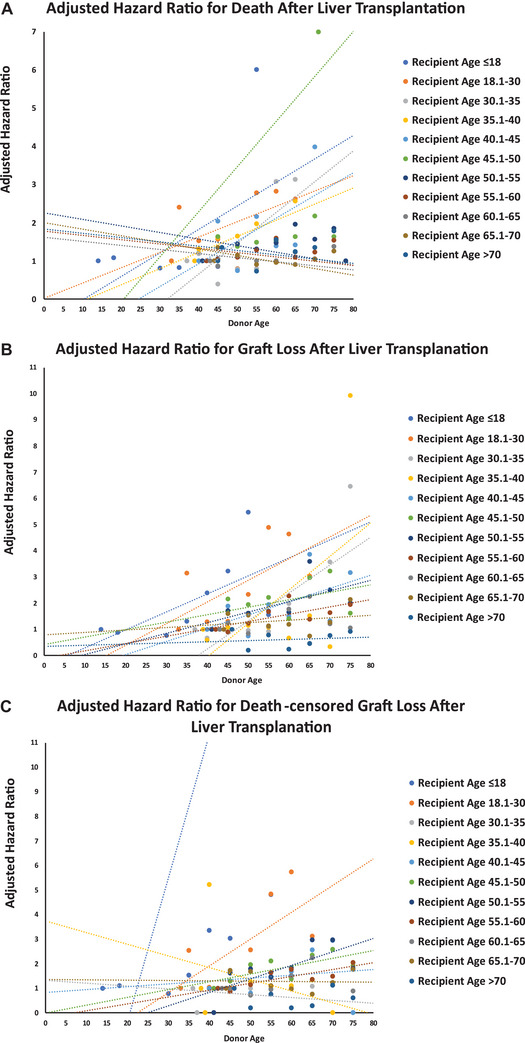
Adjusted hazard ratios (aHRs) plotted as regression lines for: (A) mortality, (B) graft loss, and (C) death‐censored graft loss, based on donor–recipient age combinations. All donor age*recipient age interaction terms were statistically significant (*p* < 0.001), indicating that the impact of increasing donor age varied substantially by recipient age (as shown by significant differences in the slopes of each regression line).

The interactions between donor and recipient age were statistically significant for overall survival, graft survival, and death‐censored graft survival, (All *p* values for interaction < 0.001). The negative impact of increasing donor age on postoperative survival was stronger in younger recipients compared to older ones (Figures 8A–C).

#### 3.2.4 Sensitivity Analysis

Excluding recipients of living donor and DCD liver transplants did not materially change the findings. The results of Cox regression models using data from recipients of DBD liver only are reported in Supplementary Tables 3, 4 and 5.

Older donor age remained significantly associated with increased mortality among pediatric recipients (aHR for donors 40.1–45 years, 3.00; 95% CI, 1.64–5.47; *p* < 0.001) and with graft loss among younger adults, including those aged 18.1–30 years (aHR for donors >60 years, 2.26; 95% CI, 1.43–3.58; *p* < 0.001). Patterns in death‐censored graft loss were similar, with donor age >65 years associated with increased risk in recipients aged 35.1–60 years. Among recipients older than 70 years, donor age was not significantly associated with outcomes, reinforcing the age‐related interaction observed in the primary analysis.

## 4 Discussion

In this national study of more than 70 000 first‐time LT recipients in the U.S., we found that the effect of donor age on posttransplant outcomes varies markedly by recipient age [31]. While older donor age was associated with significantly higher mortality and graft loss in younger recipients, its impact was attenuated among recipients aged 65 years or older. These findings confirm and extend prior reports suggesting that older donor livers can be used safely in older patients without compromising long‐term outcomes [8, 9, 10, 11, 13, 20, 32, 33].

As the proportion of older donors continues to rise, including an increasing number of individuals over age 60, this interaction between donor and recipient age has direct implications for clinical practice. Historically, older grafts were linked to poor outcomes, especially in recipients with advanced liver disease or portal hypertension [8, 9, 13, 34]. However, improvements in perioperative management, donor‐recipient matching, and organ preservation have challenged this assumption, demonstrating that older grafts can yield excellent outcomes in well‐selected recipients [10, 11]. Our analysis adds granularity by identifying specific recipient age strata and donor age thresholds where risk becomes clinically relevant.

Notably, pediatric recipients experienced a nearly threefold increase in mortality when receiving grafts from donors aged 40 to 45 years (aHR, 3.00; 95% CI, 1.64–5.47), and nearly sixfold higher risk in the primary analysis for donors over 50 years (aHR, 6.47; 95% CI, 1.92–21.83). Conversely, among recipients older than 65 years, donor age was not independently associated with survival or graft loss in adjusted models. These patterns were consistent across overall survival, graft survival, and death‐censored graft survival, and were robust to sensitivity analyses excluding LDLT and DCD grafts.

This age‐dependent effect likely reflects differences in competing mortality risks. In younger patients, long‐term graft durability is critical, and any functional limitations associated with older livers may be magnified over time [11, 12, 18, 31, 32, 33, 34]. In older recipients, limited life expectancy and competing risks of death may mitigate the impact of graft aging [6]. These results support a nuanced understanding of organ quality that goes beyond age alone and emphasize the importance of matching graft characteristics with recipient needs and projected survival.

The implications for organ allocation are substantial. Currently, donor‐recipient age matching is not explicitly incorporated into U.S. allocation policy. Our findings argue for more sophisticated, individualized approaches—minimizing extreme age mismatches in younger recipients while using older grafts more liberally in older patients. Such a strategy could safely expand access for older candidates, reduce unnecessary organ nonuse, and improve overall system efficiency.

Our study has several strengths, including the use of a large national dataset, recipient age‐stratified modeling, and validation through sensitivity analyses. However, limitations include the retrospective design, potential residual confounding, and the possibility of era effects across the 2011–2021 study window. During this period, major policy and practice changes, most notably the implementation of the AC in 2020, altered organ‐sharing patterns, geographic access, and ischemia times. We modeled transplant year as a linear covariate to partially account for secular trends, but residual era‐related confounding may persist and should be examined in future work.

In addition, SRTR does not capture several important determinants of graft quality, including steatosis, histologic finding, and detailed perfusion parameters beyond routinely recorded CIT and WIT, nor does it provide granular data on post‐transplant complications. Machine perfusion use (normothermic or hypothermic) is also not systematically coded. Given that perfusion technologies have been adopted preferentially for higher‐risk and older grafts in recent years, our inability to adjust for perfusion may have attenuated or modified the observed associations between donor age and outcomes, particularly in the later era. Finally, we used median imputation for missing covariates. Although this approach avoided case‐wise deletion and was applied in the context of a low proportion of missing data, it does not incorporate the uncertainty around missing values and may underestimate variance compared with multiple imputation. The stability of our findings in sensitivity analyses suggests that these limitations are unlikely to account fully for the observed age‐dependent risk gradients but they should be considered when interpreting our results.

Looking forward, donor age may become less central in organ selection as machine perfusion technologies mature. Normothermic and hypothermic perfusion allow functional assessment and reconditioning of marginal grafts, potentially decoupling graft viability from chronological age. Future registry and prospective studies that incorporate standardized perfusion variables will be essential to determine whether age‐based thresholds identified in the current analysis remain valid in an era of routine machine perfusion and to assess how perfusion may modify the interaction between donor and recipient age. In this evolving context, integrating functional data with recipient‐specific risk profiles and survival projections will be essential for optimizing outcomes.

## 5 Conclusions

Donor age affects LT outcomes differently across recipient ages: older grafts increase risk in younger recipients but are well tolerated in older adults. As the U.S. transplant population and donor pool both age, routinely discarding older organs is no longer justifiable. These findings support a shift toward more individualized, age‐informed allocation strategies—integrating organ preservation advances and survival benefit assessments—to make better use of available grafts and expand access for older candidates.

## Author Contributions

**Abiha Abdullah**: study design, statistical analysis, writing of the manuscript. **Berkay Demirors**: study design, statistical analysis, writing of the manuscript. **Francis Spitz**: study design, statistical analysis, writing of the manuscript. **Jason Mial‐Anthony**: study design, statistical analysis, manuscript writing. **Vrishketan Sethi**: study design, data interpretation Charbel Elias: study design, statistical analysis, writing of the manuscript. **Xingyu Zhang**: study design, data interpretation, editing of the manuscript. **Stalin Dharmayan**: data interpretation, editing of the manuscript. **Hao Liu**: study design, data interpretation. **Christopher Kaltenmeier**: study design, data interpretation. **Han Shwe**: data interpretation, editing of the manuscript. **Timothy Fokken**: data interpretation, editing of the manuscript. **Michele Molinari**: study design, data collection, statistical analysis, data interpretation and editing of the manuscript.

## Conflicts of Interest

The authors declare no conflict of interest.

## Funding

The authors have nothing to report.

## Supporting information




**Supplementary Table S1**. Distribution of donor age across recipient age categories. **Supplementary Table S2**. Missing values. **Supplementary Table S3**. Adjusted hazard ratios (aHR) for death after liver transplant categorized by donor‐recipient age groups. The aHRs were calculated using recipients of organs from donors with age equal or below the median age as the reference group. **Supplementary Table S4**. Adjusted hazard ratios (aHR) for graft loss, categorized by donor‐recipient age groups. The aHRs were calculated using recipients of organs from donors aged at or below the median age as the reference group. **Supplementary Table S5**. Adjusted hazard ratios (aHR) for death‐censored graft loss, categorized by donor‐recipient age groups. The aHRs were calculated using recipients of organs from donors aged at or below the median age as the reference group.

## Data Availability

The data that support the findings of this study are available on request from the corresponding author. The data are not publicly available due to privacy or ethical restrictions.
