# Supplementary material for: Tailoring Liver Transplant Decisions: How Donor–Recipient Age Matching Influences Outcomes
Source: Clin Transplant. 2026 Feb 15;40(2):e70477. doi: 10.1111/ctr.70477 (PMC12906856; doi:10.1111/ctr.70477)
Supplement: Supplementary file 1 — Supplementary Table S1. Distribution of donor age across recipient age categories. Supplementary Table S2. Missing values. Supplementary Table S3. Adjusted hazard ratios (aHR) for death after liver transplant categorized by donor‐recipient age groups. The aHRs were calculated using recipients of organs from donors with age equal or below the median age as the reference group. Supplementary Table S4. Adjusted hazard ratios (aHR) for graft loss, categorized by donor‐recipient age groups. The aHRs were calculated using recipients of organs from donors aged at or below the median age as the reference group. Supplementary Table S5. Adjusted hazard ratios (aHR) for death‐censored graft loss, categorized by donor‐recipient age groups. The aHRs were calculated using recipients of organs from donors aged at or below the median age as the reference group. [file CTR-40-e70477-s001.docx]

**Supplementary Material**

**Table S1.** Distribution of donor age across recipient age categories.

| **Donor Age Group** | | **Recipient Age Group** | | | | | | | | **Total** |
| --- | --- | --- | --- | --- | --- | --- | --- | --- | --- | --- |
|  |  | **0-18 years** | **18.1-30 years** | **30.1-40 years** | **40.1-50 years** | **50.1-60 years** | **60.65 years** | **65.1-70 years** | **>70 years** |  |
| **0-18 years** | Number of patients | 3,327 | 251 | 296 | 609 | 1,302 | 740 | 512 | 113 | 7,150 |
|  | % within Donor Age group | 46.5% | 3.5% | 4.1% | 8.5% | 18.2% | 10.3% | 7.2% | 1.6% | 100% |
|  | % within Recipient age group by 10 | 66.3% | 11.2% | 6.5% | 6.1% | 5.4% | 5.4% | 5.8% | 5.8% | 10.2% |
|  | % of Total | 4.7% | 0.4% | 0.4% | 0.9% | 1.9% | 1.1% | 0.7% | 0.2% | 10.2% |
| **18.1-30 years** | Number of patients | 919 | 741 | 1,253 | 2,512 | 5,581 | 2,921 | 1,679 | 367 | 15,973 |
|  | % within Donor Age group | 5.8% | 4.6% | 7.8% | 15.7% | 34.9% | 18.3% | 10.5% | 2.3% | 100% |
|  | % within Recipient age group | 18.3% | 33.1% | 27.5% | 25.0% | 23.3% | 21.5% | 19.2% | 18.9% | 22.8% |
|  | % of Total | 1.3% | 1.1% | 1.8% | 3.6% | 8.0% | 4.2% | 2.4% | 0.5% | 22.8% |
| **30.1-40 years** | Number of patients | 492 | 449 | 1,069 | 1,924 | 4,438 | 2,576 | 1,606 | 321 | 12,875 |
|  | % within Donor Age group | 3.8% | 3.5% | 8.3% | 14.9% | 34.5% | 20.0% | 12.5% | 2.5% | 100% |
|  | % within Recipient age group | 9.8% | 20.0% | 23.5% | 19.1% | 18.6% | 19.0% | 18.3% | 16.6% | 18.4% |
|  | % of Total | 0.7% | 0.6% | 1.5% | 2.7% | 6.3% | 3.7% | 2.3% | 0.5% | 18.4% |
| **40.1-50 years** | Number of patients | 204 | 409 | 893 | 2,052 | 4,518 | 2,516 | 1,621 | 322 | 12,535 |
|  | % within Donor Age group | 1.6% | 3.3% | 7.1% | 16.4% | 36.0% | 20.1% | 12.9% | 2.6% | 100% |
|  | % within Recipient age group | 4.1% | 18.2% | 19.6% | 20.4% | 18.9% | 18.5% | 18.5% | 16.6% | 17.9% |
|  | % of Total | 0.3% | 0.6% | 1.3% | 2.9% | 6.4% | 3.6% | 2.3% | 0.5% | 17.9% |
| **50.1-60 years** | Number of patients | 67 | 290 | 720 | 1,855 | 4,897 | 2,664 | 1,704 | 410 | 12,607 |
|  | % within Donor Age group | 0.5% | 2.3% | 5.7% | 14.7% | 38.8% | 21.1% | 13.5% | 3.3% | 100% |
|  | % within Recipient age group | 1.3% | 12.9% | 15.8% | 18.5% | 20.5% | 19.6% | 19.4% | 21.1% | 18.0% |
|  | % of Total | 0.1% | 0.4% | 1.0% | 2.6% | 7.0% | 3.8% | 2.4% | 0.6% | 18.0% |
| **60.65 years** | Number of patients | 6 | 57 | 188 | 609 | 1,553 | 953 | 681 | 147 | 4,194 |
|  | % within Donor Age group | 0.1% | 1.4% | 4.5% | 14.5% | 37.0% | 22.7% | 16.2% | 3.5% | 100% |
|  | % within Recipient age group | 0.1% | 2.5% | 4.1% | 6.1% | 6.5% | 7.0% | 7.8% | 7.6% | 6.0% |
|  | % of Total | 0.0% | 0.1% | 0.3% | 0.9% | 2.2% | 1.4% | 1.0% | 0.2% | 6.0% |
| **65.1-70 years** | Number of patients | 4 | 24 | 84 | 305 | 927 | 655 | 465 | 125 | 2,589 |
|  | % within Donor Age group by 10 | 0.2% | 0.9% | 3.2% | 11.8% | 35.8% | 25.3% | 18.0% | 4.8% | 100% |
|  | % within Recipient age group by 10 | 0.1% | 1.1% | 1.8% | 3.0% | 3.9% | 4.8% | 5.3% | 6.4% | 3.7% |
|  | % of Total | 0.0% | 0.0% | 0.1% | 0.4% | 1.3% | 0.9% | 0.7% | 0.2% | 3.7% |
| **>70 years** | Number of patients | 1 | 21 | 47 | 184 | 706 | 554 | 494 | 134 | 2,141 |
|  | % within Donor Age group | 0.0% | 1.0% | 2.2% | 8.6% | 33.0% | 25.9% | 23.1% | 6.3% | 100% |
|  | % within Recipient age group | 0.0% | 0.9% | 1.0% | 1.8% | 3.0% | 4.1% | 5.6% | 6.9% | 3.1% |
|  | % of Total | 0.0% | 0.0% | 0.1% | 0.3% | 1.0% | 0.8% | 0.7% | 0.2% | 3.1% |
| **Total** | Number of patients | 5,020 | 2,242 | 4,550 | 10,050 | 23,922 | 13,579 | 8,762 | 1,939 | 70,064 |
|  | % within Donor Age group | 7.2% | 3.2% | 6.5% | 14.3% | 34.1% | 19.4% | 12.5% | 2.8% | 100% |
|  | % within Recipient age group by 10 | 100.0% | 100.0% | 100.0% | 100.0% | 100.0% | 100.0% | 100.0% | 100.0% | 100% |
|  | % of Total | 7.2% | 3.2% | 6.5% | 14.3% | 34.1% | 19.4% | 12.5% | 2.8% | 100% |

| **Table S2.** Missing values | |  |
| --- | --- | --- |
| **Characteristics, n. (%)** | **Recipients, n. 70,078 (100%)** |  |
| **Age,** | 0 |  |
| **Sex** | 0 |  |
| **Blood group** | 0 |  |
| **Body Mass Index** | 1,484 (2.1%) |  |
| **MELD-Na score** | 3 (0.004%) |  |
| **History of diabetes** | 126 (0.1%) |  |
| **Need for dialysis before transplantation** | 76 (0.1%) |  |
| **Primary indication for liver transplant** | 0 |  |
| **Donor age** | 0 |  |
| **Donor sex** | 0 |  |
| **Cold ischemia time** | 1338 (1.9%) |  |
| **Warm ischemia time** | 51339 (73.2%) |  |
| **Living donor** | 0 |  |
| **Donation after cardiocirculatory arrest** | 0 |  |
| **Donation after brain death** | 0 |  |
| **Donor's primary cause of death** | 3,829 (5.4%) |  |
| **Split liver versus whole liver graft** | 26 (0.03%) |  |
|  |  |  |

**Table S3.** Adjusted hazard ratios (aHR) for death after liver transplant categorized by donor-recipient age groups. The aHRs were calculated using recipients of organs from donors with age equal or below the median age as the reference group.

| **Recipient Age (Years)** | **Donor Age (Years)** | **n. Patients** | **aHR** | **95% LCI** | **95% UCI** | | **P Value** |
| --- | --- | --- | --- | --- | --- | --- | --- |
| **0-18**  **(Median Donor Age: 14)** | **≤ 14** | **4,036** | **Reference** | | | | |
|  | **14.1-18** | **33** | **1.661** | **0.619** | **4.455** | **0.313** | |
|  | **18.1-30** | **104** | **1.301** | **0.693** | **2.443** | **0.412** | |
|  | **30.1-35** | **77** | **1.324** | **0.656** | **2.671** | **0.433** | |
|  | **35.1-40** | **38** | **2.371** | **1.121** | **5.017** | **0.024** | |
|  | **40.1-45** | **50** | **2.997** | **1.642** | **5.470** | **<0.001** | |
|  | **45.1-50** | **0** | **-** | **-** | **-** | **-** | |
|  | **>50** | **0** | **-** | **-** | **-** | **-** | |
|  | **Missing** | **0** | **-** | **-** | **-** | **-** | |
|  | **Total** | **4,338** | **-** | **-** | **-** | **-** | |
| **18.1-30**  **(Median Donor Age: 34)** | **≤ 34** | **943** | **Reference** | | | | |
|  | **34.1-35** | **75** | **1.307** | **0.683** | **2.500** | **0.418** | |
|  | **35.1-40** | **183** | **1.332** | **0.873** | **2.030** | **0.183** | |
|  | **40.1-45** | **180** | **1.481** | **0.982** | **2.232** | **0.061** | |
|  | **45.1-50** | **174** | **1.115** | **0.705** | **1.764** | **0.641** | |
|  | **50.1-55** | **150** | **1.651** | **1.089** | **2.502** | **0.018** | |
|  | **55.1-60** | **94** | **1.624** | **0.974** | **2.709** | **0.063** | |
|  | **>60** | **115** | **2.267** | **1.504** | **3.416** | **<0.001** | |
|  | **Missing** | **0** | **-** | **-** | **-** | **-** | |
|  | **Total** | **1,914** | **-** | **-** | **-** | **-** | |
| **30.1-35**  **(Median Donor Age: 36)** | **≤ 36** | **877** | **Reference** | | | | |
|  | **36.1-40** | **102** | **1.357** | **0.740** | **2.488** | **0.324** | |
|  | **40.1-45** | **147** | **0.926** | **0.516** | **1.664** | **0.797** | |
|  | **45.1-50** | **163** | **0.992** | **0.588** | **1.673** | **0.975** | |
|  | **50.1-55** | **157** | **0.996** | **0.574** | **1.728** | **0.989** | |
|  | **55.1-60** | **93** | **1.518** | **0.828** | **2.784** | **0.177** | |
|  | **>60** | **130** | **2.054** | **1.293** | **3.265** | **0.002** | |
|  | **Missing** | **0** | **-** | **-** | **-** | **-** | |
|  | **Total** | **1,669** | **-** | **-** | **-** | **-** | |
| **35.1-40**  **(Median Donor Age: 39)** | **≤ 39** | **1,130** | **Reference** | | | | |
|  | **39.1-40** | **86** | **1.513** | **0.812** | **2.818** | **0.192** | |
|  | **40.1-45** | **218** | **1.677** | **1.127** | **2.496** | **0.011** | |
|  | **45.1-50** | **251** | **1.765** | **1.224** | **2.546** | **0.002** | |
|  | **50.1-55** | **240** | **1.615** | **1.099** | **2.371** | **0.015** | |
|  | **55.1-60** | **184** | **1.831** | **1.224** | **2.739** | **0.003** | |
|  | **60.1-65** | **118** | **1.734** | **1.050** | **2.864** | **0.031** | |
|  | **>65** | **109** | **2.062** | **1.289** | **3.300** | **0.003** | |
|  | **Missing** | **21** | **-** | **-** | **-** | **-** | |
|  | **Total** | **2,357** | **-** | **-** | **-** | **-** | |
| **40.1-45**  **(Median Donor Age: 40)** | **≤ 40** | **1,610** | **Reference** | | | | |
|  | **40.1-45** | **288** | **1.049** | **0.722** | **1.522** | **0.803** | |
|  | **45.1-50** | **317** | **0.806** | **0.547** | **1.188** | **0.276** | |
|  | **50.1-55** | **326** | **1.439** | **1.059** | **1.953** | **0.020** | |
|  | **55.1-60** | **309** | **1.143** | **0.807** | **1.618** | **0.452** | |
|  | **60.1-65** | **224** | **1.378** | **0.949** | **2.000** | **0.092** | |
|  | **65.1-70** | **117** | **1.136** | **0.670** | **1.926** | **0.636** | |
|  | **>70** | **76** | **2.151** | **1.339** | **3.455** | **0.002** | |
|  | **Missing** | **24** | **-** | **-** | **-** | **-** | |
|  | **Total** | **3,291** | **-** | **-** | **-** | **-** | |
| **45.1-50**  **(Median Donor Age: 41)** | **≤ 41** | **2,712** | **Reference** | | | | |
|  | **41.1-45** | **381** | **1.272** | **0.962** | **1.682** | **0.091** | |
|  | **45.1-50** | **558** | **1.428** | **1.138** | **1.791** | **0.002** | |
|  | **50.1-55** | **543** | **1.213** | **0.955** | **1.540** | **0.113** | |
|  | **55.1-60** | **558** | **1.227** | **0.963** | **1.564** | **0.097** | |
|  | **60.1-65** | **414** | **1.324** | **1.015** | **1.728** | **0.039** | |
|  | **65.1-70** | **231** | **1.653** | **1.214** | **2.251** | **0.001** | |
|  | **>70** | **141** | **1.383** | **0.921** | **2.077** | **0.118** | |
|  | **Missing** | **32** | **-** | **-** | **-** | **-** | |
|  | **Total** | **5,570** | **-** | **-** | **-** | **-** | |
| **50.1-55**  **(Median Donor Age: 43)** | **≤ 43** | **4,380** | **Reference** | | | | |
|  | **43.1-45** | **308** | **1.198** | **0.903** | **1.590** | **0.210** | |
|  | **45.1-50** | **876** | **1.254** | **1.054** | **1.492** | **0.011** | |
|  | **50.1-55** | **992** | **1.311** | **1.115** | **1.541** | **0.001** | |
|  | **55.1-60** | **903** | **1.341** | **1.130** | **1.593** | **<0.001** | |
|  | **60.1-65** | **624** | **1.367** | **1.122** | **1.666** | **0.002** | |
|  | **65.1-70** | **411** | **1.327** | **1.040** | **1.694** | **0.023** | |
|  | **>70** | **310** | **1.515** | **1.175** | **1.952** | **0.001** | |
|  | **Missing** | **75** | **-** | **-** | **-** | **-** | |
|  | **Total** | **8,879** | **-** | **-** | **-** | **-** | |
| **55.1-60**  **(Median Donor Age: 44)** | **≤44** | **6,138** | **Reference** | | | | |
|  | **44.1-45** | **205** | **1.031** | **0.743** | **1.431** | **0.853** | |
|  | **45.1-50** | **1,158** | **1.013** | **0.872** | **1.176** | **0.867** | |
|  | **50.1-55** | **1,363** | **1.116** | **0.975** | **1.277** | **0.112** | |
|  | **55.1-60** | **1,227** | **1.391** | **1.220** | **1.585** | **<0.001** | |
|  | **60.1-65** | **965** | **1.101** | **0.939** | **1.291** | **0.235** | |
|  | **65.1-70** | **612** | **1.168** | **0.966** | **1.413** | **0.109** | |
|  | **>70** | **482** | **1.322** | **1.083** | **1.614** | **0.006** | |
|  | **Missing** | **121** | **-** | **-** | **-** | **-** | |
|  | **Total** | **12,271** | **-** | **-** | **-** | **-** | |
| **60.1-65**  **(Median Donor Age: 45)** | **≤45** | **5,971** | **Reference** | | | | |
|  | **45.1-50** | **1,113** | **0.992** | **0.859** | **1.145** | **0.908** | |
|  | **50.1-55** | **1,213** | **1.028** | **0.896** | **1.178** | **0.696** | |
|  | **55.1-60** | **1,215** | **1.062** | **0.927** | **1.217** | **0.383** | |
|  | **60.1-65** | **967** | **1.033** | **0.888** | **1.202** | **0.671** | |
|  | **65.1-70** | **702** | **1.152** | **0.975** | **1.360** | **0.096** | |
|  | **>70** | **625** | **1.112** | **0.929** | **1.333** | **0.247** | |
|  | **Missing** | **85** | **-** | **-** | **-** | **-** | |
|  | **Total** | **11,891** | **-** | **-** | **-** | **-** | |
| **65.1-70**  **(Median Donor Age: 46)** | **≤ 46** | **3,755** | **Reference** | | | | |
|  | **46.1-50** | **546** | **0.980** | **0.805** | **1.192** | **0.837** | |
|  | **50.1-55** | **785** | **0.959** | **0.809** | **1.137** | **0.631** | |
|  | **55.1-60** | **747** | **0.737** | **1.030** | **0.868** | **1.222** | |
|  | **60.1-65** | **658** | **0.389** | **1.083** | **0.904** | **1.297** | |
|  | **65.1-70** | **496** | **0.796** | **1.083** | **0.904** | **1.297** | |
|  | **>70** | **542** | **0.369** | **1.092** | **0.901** | **1.325** | |
|  | **Missing** | **72** | **-** | **-** | **-** | **-** | |
|  | **Total** | **7,601** | **-** | **-** | **-** | **-** | |
| **>70**  **(Median Donor Age: 48)** | **≤ 48** | **787** | **Reference** | | | | |
|  | **48.1-50** | **93** | **1.388** | **0.931** | **2.067** | **0.107** | |
|  | **50.1-55** | **163** | **0.987** | **0.697** | **1.396** | **0.939** | |
|  | **55.1-60** | **182** | **1.108** | **0.797** | **1.541** | **0.542** | |
|  | **60.1-65** | **142** | **1.401** | **1.001** | **1.961** | **0.050** | |
|  | **65.1-70** | **130** | **0.963** | **0.647** | **1.435** | **0.854** | |
|  | **>70** | **147** | **0.990** | **0.679** | **1.442** | **0.958** | |
|  | **Missing** | **19** | **-** | **-** | **-** | **-** | |
|  | **Total** | **1,663** | **-** | **-** | **-** | **-** | |

**Legend:** Adjusted hazard ratios (aHR) were determined using multivariate Cox regression models adjusted for recipient characteristics (sex, BMI, blood group, race/ethnicity, history of diabetes, history of dialysis, primary indication for LT, functional status, and MELD-Na score), donor characteristics (sex, primary cause of death, race/ethnicity, BMI, and organ characteristics (whole organ or split organ), as well as the year of transplantation, warm ischemia time (WIT), and cold ischemia time (CIT).

**Table S4.** Adjusted hazard ratios (aHR) for graft loss, categorized by donor-recipient age groups. The aHRs were calculated using recipients of organs from donors aged at or below the median age as the reference group.

| **Recipient Age (Years)** | **Donor Age (Years)** | **n. Patients** | **aHR** | **95% LCI** | **95% UCI** | | **P Value** |
| --- | --- | --- | --- | --- | --- | --- | --- |
| **0-18**  **(Median Donor Age: 14)** | **≤ 14** | **4,036** | **Reference** | | | | |
|  | **14.1-18** | **33** | **2.233** | **1.058** | **4.713** | **0.035** | |
|  | **18.1-30** | **104** | **1.058** | **0.581** | **1.925** | **0.855** | |
|  | **30.1-35** | **77** | **1.518** | **0.855** | **2.695** | **0.154** | |
|  | **35.1-40** | **38** | **2.917** | **1.603** | **5.309** | **<0.001** | |
|  | **40.1-45** | **50** | **1.813** | **0.937** | **3.509** | **0.077** | |
|  | **45.1-50** | **0** | **-** | **-** | **-** | **-** | |
|  | **>50** | **0** | **-** | **-** | **-** | **-** | |
|  | **Missing** | **0** | **-** | **-** | **-** | **-** | |
|  | **Total** | **4,338** | **-** | **-** | **-** | **-** | |
| **18.1-30**  **(Median Donor Age: 34)** | **≤ 34** | **943** | **Reference** | | | | |
|  | **34.1-35** | **75** | **0.936** | **0.409** | **2.140** | **0.875** | |
|  | **35.1-40** | **183** | **0.817** | **0.465** | **1.435** | **0.483** | |
|  | **40.1-45** | **180** | **1.031** | **0.614** | **1.732** | **0.907** | |
|  | **45.1-50** | **174** | **1.667** | **1.084** | **2.653** | **0.020** | |
|  | **50.1-55** | **150** | **1.879** | **1.214** | **2.907** | **0.005** | |
|  | **55.1-60** | **94** | **2.055** | **1.224** | **3.453** | **0.006** | |
|  | **>60** | **115** | **2.264** | **1.431** | **3.581** | **<0.001** | |
|  | **Missing** | **0** | **-** | **-** | **-** | **-** | |
|  | **Total** | **1,914** | **-** | **-** | **-** | **-** | |
| **30.1-35**  **(Median Donor Age: 36)** | **≤ 36** | **877** | **Reference** | | | | |
|  | **36.1-40** | **102** | **0.799** | **0.347** | **1.842** | **0.598** | |
|  | **40.1-45** | **147** | **0.973** | **0.514** | **1.842** | **0.934** | |
|  | **45.1-50** | **163** | **0.959** | **0.530** | **1.738** | **0.891** | |
|  | **50.1-55** | **157** | **1.696** | **1.029** | **2.794** | **0.038** | |
|  | **55.1-60** | **93** | **1.351** | **0.674** | **2.710** | **0.396** | |
|  | **>60** | **130** | **2.339** | **1.432** | **3.819** | **<0.001** | |
|  | **Missing** | **0** | **-** | **-** | **-** | **-** | |
|  | **Total** | **1,669** | **-** | **-** | **-** | **-** | |
| **35.1-40**  **(Median Donor Age: 39)** | **≤ 39** | **1,130** | **Reference** | | | | |
|  | **39.1-40** | **86** | **2.947** | **1.586** | **5.478** | **<0.001** | |
|  | **40.1-45** | **218** | **1.827** | **1.101** | **3.031** | **0.020** | |
|  | **45.1-50** | **251** | **1.509** | **0.909** | **2.503** | **0.111** | |
|  | **50.1-55** | **240** | **1.628** | **0.990** | **2.676** | **0.055** | |
|  | **55.1-60** | **184** | **2.030** | **1.224** | **3.368** | **0.006** | |
|  | **60.1-65** | **118** | **1.649** | **0.844** | **3.220** | **0.143** | |
|  | **>65** | **109** | **3.290** | **1.964** | **5.513** | **<0.001** | |
|  | **Missing** | **21** | **-** | **-** | **-** | **-** | |
|  | **Total** | **2,357** | **-** | **-** | **-** | **-** | |
| **40.1-45**  **(Median Donor Age: 40)** | **≤ 40** | **1,610** | **Reference** | | | | |
|  | **40.1-45** | **288** | **1.046** | **0.632** | **1.730** | **0.862** | |
|  | **45.1-50** | **317** | **1.159** | **0.735** | **1.826** | **0.526** | |
|  | **50.1-55** | **326** | **1.649** | **1.110** | **2.449** | **0.013** | |
|  | **55.1-60** | **309** | **1.449** | **0.945** | **2.221** | **0.089** | |
|  | **60.1-65** | **224** | **1.677** | **1.055** | **2.664** | **0.029** | |
|  | **65.1-70** | **117** | **1.400** | **0.730** | **2.684** | **0.312** | |
|  | **>70** | **76** | **2.510** | **1.377** | **4.574** | **0.003** | |
|  | **Missing** | **24** | **-** | **-** | **-** | **-** | |
|  | **Total** | **3,291** | **-** | **-** | **-** | **-** | |
| **45.1-50**  **(Median Donor Age: 41)** | **≤ 41** | **2,712** | **Reference** | | | | |
|  | **41.1-45** | **381** | **1.227** | **0.789** | **1.909** | **0.364** | |
|  | **45.1-50** | **558** | **1.555** | **1.103** | **2.191** | **0.012** | |
|  | **50.1-55** | **543** | **1.780** | **1.284** | **2.468** | **<0.001** | |
|  | **55.1-60** | **558** | **1.459** | **1.025** | **2.076** | **0.036** | |
|  | **60.1-65** | **414** | **2.235** | **1.595** | **3.132** | **<0.001** | |
|  | **65.1-70** | **231** | **2.499** | **1.663** | **3.754** | **<0.001** | |
|  | **>70** | **141** | **2.081** | **1.221** | **3.548** | **0.007** | |
|  | **Missing** | **32** | **-** | **-** | **-** | **-** | |
|  | **Total** | **5,570** | **-** | **-** | **-** | **-** | |
| **50.1-55**  **(Median Donor Age: 43)** | **≤ 43** | **4,380** | **Reference** | | | | |
|  | **43.1-45** | **308** | **1.037** | **0.625** | **1.723** | **0.888** | |
|  | **45.1-50** | **876** | **1.457** | **1.103** | **1.924** | **0.008** | |
|  | **50.1-55** | **992** | **1.385** | **1.057** | **1.814** | **0.018** | |
|  | **55.1-60** | **903** | **1.593** | **1.218** | **2.085** | **<0.001** | |
|  | **60.1-65** | **624** | **2.062** | **1.559** | **2.728** | **<0.001** | |
|  | **65.1-70** | **411** | **1.975** | **1.405** | **2.775** | **<0.001** | |
|  | **>70** | **310** | **3.004** | **2.180** | **4.138** | **<0.001** | |
|  | **Missing** | **75** | **-** | **-** | **-** | **-** | |
|  | **Total** | **8,879** | **-** | **-** | **-** | **-** | |
| **55.1-60**  **(Median Donor Age: 44)** | **≤44** | **6,138** | **Reference** | | | | |
|  | **44.1-45** | **205** | **1.647** | **1.011** | **2.683** | **0.045** | |
|  | **45.1-50** | **1,158** | **1.022** | **0.776** | **1.348** | **0.875** | |
|  | **50.1-55** | **1,363** | **1.776** | **1.440** | **2.191** | **<0.001** | |
|  | **55.1-60** | **1,227** | **1.504** | **1.191** | **1.899** | **<0.001** | |
|  | **60.1-65** | **965** | **1.364** | **1.044** | **1.782** | **0.023** | |
|  | **65.1-70** | **612** | **1.539** | **1.129** | **2.097** | **0.006** | |
|  | **>70** | **482** | **1.882** | **1.373** | **2.581** | **<0.001** | |
|  | **Missing** | **121** | **-** | **-** | **-** | **-** | |
|  | **Total** | **12,271** | **-** | **-** | **-** | **-** | |
| **60.1-65**  **(Median Donor Age: 45)** | **≤45** | **5,971** | **Reference** | | | | |
|  | **45.1-50** | **1,113** | **1.098** | **0.822** | **1.467** | **0.528** | |
|  | **50.1-55** | **1,213** | **1.267** | **0.973** | **1.651** | **0.079** | |
|  | **55.1-60** | **1,215** | **1.502** | **1.172** | **1.926** | **0.001** | |
|  | **60.1-65** | **967** | **1.519** | **1.159** | **1.992** | **0.002** | |
|  | **65.1-70** | **702** | **1.334** | **0.963** | **1.864** | **0.083** | |
|  | **>70** | **625** | **1.821** | **1.347** | **2.463** | **<0.001** | |
|  | **Missing** | **85** | **-** | **-** | **-** | **-** | |
|  | **Total** | **11,891** | **-** | **-** | **-** | **-** | |
| **65.1-70**  **(Median Donor Age: 46)** | **≤ 46** | **3,755** | **Reference** | | | | |
|  | **46.1-50** | **546** | **1.262** | **0.837** | **1.903** | **0.266** | |
|  | **50.1-55** | **785** | **1.402** | **0.998** | **1.969** | **0.052** | |
|  | **55.1-60** | **747** | **1.351** | **0.949** | **1.923** | **0.095** | |
|  | **60.1-65** | **658** | **1.569** | **1.102** | **2.234** | **0.013** | |
|  | **65.1-70** | **496** | **1.563** | **1.055** | **2.316** | **0.026** | |
|  | **>70** | **542** | **1.764** | **1.225** | **2.540** | **0.002** | |
|  | **Missing** | **72** | **-** | **-** | **-** | **-** | |
|  | **Total** | **7,601** | **-** | **-** | **-** | **-** | |
| **>70**  **(Median Donor Age: 48)** | **≤ 48** | **787** | **Reference** | | | | |
|  | **48.1-50** | **93** | **0.463** | **0.112** | **1.925** | **0.290** | |
|  | **50.1-55** | **163** | **1.204** | **0.580** | **2.500** | **0.618** | |
|  | **55.1-60** | **182** | **0.845** | **0.376** | **1.899** | **0.683** | |
|  | **60.1-65** | **142** | **0.781** | **0.306** | **1.989** | **0.604** | |
|  | **65.1-70** | **130** | **1.019** | **0.429** | **2.418** | **0.966** | |
|  | **>70** | **147** | **0.907** | **0.382** | **2.152** | **0.824** | |
|  | **Missing** | **19** | **-** | **-** | **-** | **-** | |
|  | **Total** | **1,663** | **-** | **-** | **-** | **-** | |

**Legend:** Adjusted hazard ratios (aHR) were determined using multivariate Cox regression models adjusted for recipient characteristics (sex, BMI, blood group, race/ethnicity, history of diabetes, history of dialysis, primary indication for LT, functional status, and MELD-Na score), donor characteristics (sex, primary cause of death, race/ethnicity, BMI, and organ characteristics (whole organ or split organ), as well as the year of transplantation, warm ischemia time (WIT), and cold ischemia time (CIT).

**Table S5**. Adjusted hazard ratios (aHR) for death-censored graft loss, categorized by donor-recipient age groups. The aHRs were calculated using recipients of organs from donors aged at or below the median age as the reference group.

| **Recipient Age (Years)** | **Donor Age (Years)** | **n. Patients** | **aHR** | **95% LCI** | **95% UCI** | | **P Value** |
| --- | --- | --- | --- | --- | --- | --- | --- |
| ≤ 18  (Median Donor Age: 14) | ≤ 14 | 4,036 | **Reference** | | | | |
|  | **14.1-18** | **33** | **2.646** | **1.252** | **5.588** | **0.011** | |
|  | 18.1-30 | 104 | 1.155 | 0.635 | 2.103 | 0.636 | |
|  | 30.1-35 | 77 | 1.586 | 0.893 | 2.816 | 0.116 | |
|  | **35.1-40** | **38** | **2.675** | **1.469** | **4.870** | **0.001** | |
|  | 40.1-45 | 50 | 1.519 | 0.784 | 2.942 | 0.215 | |
|  | 45.1-50 | 0 | - | - | - | - | |
|  | >50 | 0 | - | - | - | - | |
|  | Missing | 0 | - | - | - | - | |
|  | **Total** | **4,338** | **-** | **-** | **-** | **-** | |
| 18.1-30  (Median Donor Age: 34) | ≤ 34 | 943 | **Reference** | | | | |
|  | 34.1-35 | 75 | 1.132 | 0.494 | 2.593 | 0.769 | |
|  | 35.1-40 | 183 | 0.815 | 0.464 | 1.432 | 0.477 | |
|  | 40.1-45 | 180 | 0.976 | 0.580 | 1.643 | 0.928 | |
|  | **45.1-50** | **174** | **1.663** | **1.082** | **2.557** | **0.020** | |
|  | **50.1-55** | **150** | **1.898** | **1.226** | **2.938** | **0.004** | |
|  | **55.1-60** | **94** | **2.106** | **1.253** | **3.540** | **0.005** | |
|  | **>60** | **115** | **2.364** | **1.493** | **3.743** | **<0.001** | |
|  | Missing | 0 | - | - | - | - | |
|  | **Total** | **1,914** | **-** | **-** | **-** | **-** | |
| 30.1-35  (Median Donor Age: 36) | ≤ 36 | 877 | **Reference** | | | | |
|  | 36.1-40 | 102 | 0.842 | 0.365 | 1.940 | 0.686 | |
|  | 40.1-45 | 147 | 0.906 | 0.478 | 1.714 | 0.761 | |
|  | 45.1-50 | 163 | 0.805 | 0.435 | 1.489 | 0.490 | |
|  | **50.1-55** | **157** | **1.579** | **0.958** | **2.602** | **0.073** | |
|  | 55.1-60 | 93 | 1.450 | 0.723 | 2.908 | 0.296 | |
|  | **>60** | **130** | **2.355** | **1.441** | **3.847** | **<0.001** | |
|  | Missing | 0 | - | - | - | - | |
|  | **Total** | **1,669** | **-** | **-** | **-** | **-** | |
| 35.1-40  (Median Donor Age: 39) | ≤ 39 | 1,130 | **Reference** | | | | |
|  | **39.1-40** | **86** | **2.729** | **1.468** | **5.072** | **0.002** | |
|  | **40.1-45** | **218** | **1.829** | **1.102** | **3.034** | **0.019** | |
|  | 45.1-50 | 251 | 1.379 | 0.831 | 2.289 | 0.214 | |
|  | 50.1-55 | 240 | 1.611 | 0.980 | 2.648 | 0.060 | |
|  | **55.1-60** | **184** | **2.000** | **1.206** | **3.319** | **0.007** | |
|  | 60.1-65 | 118 | 1.680 | 0.860 | 3.282 | 0.129 | |
|  | **>65** | **109** | **2.961** | **1.766** | **4.966** | **<0.001** | |
|  | Missing | 21 | - | - | - | - | |
|  | **Total** | **2,357** | **-** | **-** | **-** | **-** | |
| 40.1-45  (Median Donor Age: 40) | ≤ 40 | 1,610 | **Reference** | | | | |
|  | 40.1-45 | 288 | 0.996 | 0.602 | 1.647 | 0.986 | |
|  | 45.1-50 | 317 | 1.019 | 0.646 | 1.607 | 0.935 | |
|  | **50.1-55** | **326** | **1.492** | **1.004** | **2.216** | **0.048** | |
|  | 55.1-60 | 309 | 1.391 | 0.908 | 2.133 | 0.130 | |
|  | **60.1-65** | **224** | **1.599** | **1.006** | **2.542** | **0.047** | |
|  | 65.1-70 | 117 | 1.354 | 0.705 | 2.599 | 0.363 | |
|  | **>70** | **76** | **2.450** | **1.344** | **4.467** | **0.003** | |
|  | Missing | 24 | - | - | - | - | |
|  | **Total** | **3,291** | **-** | **-** | **-** | **-** | |
| 45.1-50  (Median Donor Age: 41) | ≤ 41 | 2,712 | **Reference** | | | | |
|  | 41.1-45 | 381 | 1.241 | 0.798 | 1.932 | 0.338 | |
|  | **45.1-50** | **558** | **1.531** | **1.086** | **2.157** | **0.015** | |
|  | **50.1-55** | **543** | **1.664** | **1.200** | **2.308** | **0.002** | |
|  | **55.1-60** | **558** | **1.437** | **1.010** | **2.045** | **0.044** | |
|  | **60.1-65** | **414** | **2.232** | **1.592** | **3.127** | **<0.001** | |
|  | **65.1-70** | **231** | **2.333** | **1.552** | **3.506** | **<0.001** | |
|  | **>70** | **141** | **1.913** | **1.122** | **3.263** | **0.017** | |
|  | Missing | 32 | - | - | - | - | |
|  | **Total** | **5,570** | **-** | **-** | **-** | **-** | |
| 50.1-55  (Median Donor Age: 43) | ≤ 43 | 4,380 | **Reference** | | | | |
|  | 43.1-45 | 308 | 1.085 | 0.653 | 1.802 | 0.753 | |
|  | **45.1-50** | **876** | **1.435** | **1.086** | **1.895** | **0.011** | |
|  | **50.1-55** | **992** | **1.358** | **1.036** | **1.779** | **0.027** | |
|  | **55.1-60** | **903** | **1.684** | **1.287** | **2.204** | **<0.001** | |
|  | **60.1-65** | **624** | **2.162** | **1.634** | **2.861** | **<0.001** | |
|  | **65.1-70** | **411** | **2.134** | **1.518** | **3.000** | **<0.001** | |
|  | **>70** | **310** | **3.010** | **2.185** | **4.148** | **<0.001** | |
|  | Missing | 75 | - | - | - | - | |
|  | **Total** | **8,879** | **-** | **-** | **-** | **-** | |
| 55.1-60  (Median Donor Age: 44) | ≤44 | 6,138 | **Reference** | | | | |
|  | 44.1-45 | 205 | 1.618 | 0.993 | 2.637 | 0.054 | |
|  | 45.1-50 | 1,158 | 1.020 | 0.774 | 1.344 | 0.889 | |
|  | **50.1-55** | **1,363** | **1.782** | **1.445** | **2.198** | **<0.001** | |
|  | **55.1-60** | **1,227** | **1.534** | **1.215** | **1.938** | **<0.001** | |
|  | **60.1-65** | **965** | **1.403** | **1.074** | **1.833** | **0.013** | |
|  | **65.1-70** | **612** | **1.558** | **1.143** | **2.124** | **0.005** | |
|  | **>70** | **482** | **1.894** | **1.381** | **2.597** | **<0.001** | |
|  | Missing | 121 | - | - | - | - | |
|  | **Total** | **12,271** | **-** | **-** | **-** | **-** | |
| 60.1-65  (Median Donor Age: 45) | ≤45 | 5,971 | **Reference** | | | | |
|  | 45.1-50 | 1,113 | 1.086 | 0.812 | 1.451 | 0.579 | |
|  | 50.1-55 | 1,213 | 1.251 | 0.960 | 1.630 | 0.097 | |
|  | **55.1-60** | **1,215** | **1.467** | **1.144** | **1.881** | **0.003** | |
|  | **60.1-65** | **967** | **1.491** | **1.137** | **1.955** | **0.004** | |
|  | 65.1-70 | 702 | 1.336 | 0.965 | 1.850 | 0.081 | |
|  | **>70** | **625** | **1.843** | **1.363** | **2.493** | **<0.001** | |
|  | Missing | 85 | - | - | - | - | |
|  | **Total** | **11,891** | **-** | **-** | **-** | **-** | |
| 65.1-70  (Median Donor Age: 46) | ≤ 46 | 3,755 | **Reference** | | | | |
|  | 46.1-50 | 546 | 1.248 | 0.828 | 1.881 | 0.291 | |
|  | 50.1-55 | 785 | 1.386 | 0.986 | 1.947 | 0.060 | |
|  | 55.1-60 | 747 | 1.369 | 0.961 | 1.949 | 0.082 | |
|  | **60.1-65** | **658** | **1.624** | **1.140** | **2.312** | **0.007** | |
|  | **65.1-70** | **496** | **1.554** | **1.049** | **2.303** | **0.028** | |
|  | **>70** | **542** | **1.768** | **1.228** | **2.546** | **0.002** | |
|  | Missing | 72 | - | - | - | - | |
|  | **Total** | **7,601** | **-** | **-** | **-** | **-** | |
| >70  (Median Donor Age: 48) | ≤ 48 | 787 | **Reference** | | | | |
|  | 48.1-50 | 93 | 0.488 | 0.118 | 2.028 | 0.324 | |
|  | 50.1-55 | 163 | 1.133 | 0.545 | 2.355 | 0.737 | |
|  | 55.1-60 | 182 | 0.876 | 0.390 | 1.970 | 0.749 | |
|  | 60.1-65 | 142 | 0.818 | 0.321 | 2.086 | 0.675 | |
|  | 65.1-70 | 130 | 1.029 | 0.433 | 2.442 | 0.949 | |
|  | >70 | 147 | 0.914 | 0.385 | 2.170 | 0.839 | |
|  | Missing | 19 | - | - | - | - | |
|  | **Total** | **1,663** | **-** | **-** | **-** | **-** | |

**Legend:** Adjusted hazard ratios (aHR) were determined using multivariate Cox regression models adjusted for recipient characteristics (sex, BMI, blood group, race/ethnicity, history of diabetes, history of dialysis, primary indication for LT, functional status, and MELD-Na score), donor characteristics (sex, primary cause of death, race/ethnicity, BMI, and organ characteristics (whole organ or split organ), as well as the year of transplantation, warm ischemia time (WIT), and cold ischemia time (CIT).
